# Monitoring pesticide residues in pepper (*Capsicum annuum* L.) from Al-Qassim region, Saudi Arabia: Occurrence, quality, and risk evaluations

**DOI:** 10.1016/j.heliyon.2024.e36805

**Published:** 2024-08-29

**Authors:** Fahad M. Alminderej, Sayed M. Saleh, Osama I. Abdallah

**Affiliations:** a Department of Chemistry, College of Science, Qassim University, Buraidah 51452, Saudi Arabia; b Pesticide Residues and Environmental Pollution Department, Central Agricultural Pesticide Laboratory, Agriculture Research Center, Dokki, Giza, 12618, Egypt; c Food Safety Laboratory, Al-Qassim Municipality, Buraidah, Saudi Arabia

**Keywords:** Pesticide residues, Risk assessment, Method validation, Quality index, Peppers

## Abstract

The Al-Qassim region, a prominent agricultural hub in Saudi Arabia, significantly contributes to the national production of vegetables and fruits. This study validated the standard EN-QuEChERS (Quick, Easy, Cheap, Effective, Rugged and Safe) method in conjunction with liquid chromatography–tandem mass spectrometry (LC–MS/MS) to determine 90 multiple pesticide residues in three categories of peppers: green bell, green hot and red chilli peppers. Validation criteria, including linearity range, accuracy, precision, limit of detection (LOD), and limit of quantification (LOQ), were within the acceptance range of the SANTE/11312/2021 guideline. The validated method was then used to analyse 536 pepper samples collected in 2023 from the Al-Qassim region of Saudi Arabia. The analysis of 536 pepper samples revealed that 394 samples (73.51 %) contained pesticide residues, with 126 (23.51 %) exceeding the established maximum residue limits (MRLs). The most frequently identified pesticide was imidacloprid (171 samples, 31.9 %) and acetamiprid (94 samples, 17.54 %), followed by bifenazate and difenoconazole, which were each detected in 66 samples (12.31 %). Among the remaining 32 pesticides, 24 were detected in 1%–10 % of the samples, whereas 8 were detected in <1 %. The 36 pesticides detected were classified into 14 insecticides (38.9 %), 14 fungicides (38.9 %) and 8 acaricides (22.2 %). Notably, the overall detection rate of the pesticides was relatively higher in red chilli peppers (232 %) compared with bell peppers (165 %), followed by green hot peppers (132 %). Red chilli peppers also showed the highest residue concentrations of various pesticides. Neonicotinoids and triazoles exhibited the highest detection rates in this study. The residue quality index (IqR) of the samples analysed fell into the categories excellent (26.49 %), good (31.72 %), and adequate (14.06 %), with 28.73 % of the samples deemed inadequate. Long-term dietary exposure was examined for adults and children. This study highlights the crucial role of continual observation in defending public health and securing the trade standardisation and safety.

## Abbreviations

ADI: acceptable daily intake
EDI: estimated daily intake
EFSA: European Food Safety Authority
GAP: good agricultural practice
MRL: maximum residue limit
PHI: pre‐harvest interval
PSA: primary secondary amine
QuEChERS: quick easy cheap effective rugged safe
IqR: index of quality for residues
HQc: chronic hazard quotient
HIc: hazard index
LOD: the limit of detection
LOQ: the limit of quantitation
PRC: pesticide residue content

## 1 Introduction

Pepper (*Capsicum annuum* L.) ranks among the world's most valued vegetables and spices, celebrated for its abundance of health-beneficial compounds, including anthocyanins, vitamins, phenolic acids, flavonoids, carotenoids and capsaicin [1,2]. Its benefits include lowering cholesterol levels, reducing the risk of atherosclerotic cardiovascular disease, and fighting infections, bacteria, and free radicals [[3], [4], [5], [6]].

Contamination with pesticide residues is a significant problem for fruit and vegetable crops worldwide. Such residues, which can have acute and chronic health consequences for humans, are a constant threat. As pests relentlessly invade open fields and greenhouses, farmers must use pesticides to protect their crops and prevent potential losses [7,8] Consequently, numerous countries and international bodies have introduced maximum limits on pesticide residues. This initiative aims to ensure strict adherence to good agricultural practices (GAP) and protect consumers from the risks of these chemicals.

In 2023, the European Union (EU) set MRLs for peppers covering >505 pesticides and the corresponding degradation products (EU-MRL database) [9]. In addition, the Codex Alimentarius Commission established MRLs for 24 pesticides [10], whereas Saudi Arabia introduced MRLs for 70 pesticides [11]. Recently, national safety agencies conducted a dietary pesticide intake analysis to determine whether residues in commonly consumed agricultural products pose a risk to consumers [12,13]. This assessment estimates the risks associated with consuming pesticide residues in food by combining data on consumption and contamination from monitoring initiatives. These risks are then compared with toxicological reference values, typically the acceptable daily intake (ADI) or an acute reference dose, to ensure that the residues in food are safe for consumption [14]**.**

Saudi Arabia is known for its extreme heat and humidity, and farmers often grow plants in greenhouses to protect them from harsh climatic conditions. The use of pesticides for pest control is expected to increase in these enclosed growing spaces. Studies have reported that pesticide residue degradation rates differ significantly between vegetables grown in greenhouses and those grown outdoors. For example, penthiopyrad isomers in cucumbers and tomatoes grown under field conditions exhibited faster dissipation rates (t_0.5_ = 2.51–3.90 d) than in greenhouse samples (with a half-life of 3.23–7.85 d) [15]**.**

Higher pesticide residues are often particularly found in peppers than in other agricultural products owing to the repeated application of these substances during cultivation [16]**.** Previous studies have detected pesticide residues in several types of peppers. Among the 108 pepper samples collected from Çanakkale (Turkey) public markets, 68.5 % were contaminated with pesticide residues [17]**.** According to the 2016 EU pesticide residue report on unprocessed peppers, an examination of 6451 samples of sweet peppers showed that 51 % were contaminated with pesticides [18]. In the Konya region of Turkey, 80 % of the 10 pepper samples sourced from local markets tested positive for pesticide residues [19]. Furthermore, pesticide residues were found in 68.2 % of 211 pepper samples collected from the Asir area in Saudi Arabia [20]. Another study found that 28.7 % of 299 pepper samples from wholesale markets, supermarkets, and bazaars in the Chinese province of Shandong contained residues [21]. Therefore, continuously monitoring pesticide residues in pepper products is crucial for assessing their quality and potential health risks [22,23].

Regulatory authorities require methods to detect extremely very low concentrations of pesticide residues, and the different properties of pesticides, such as polarity, volatility, and solubility, increase the method's complexity, making the development of efficient strategies for the analysis of pesticide residues considerably challenging. The co-extraction of the matrix further complicates the process [24]. To solve these problems, researchers have turned to mass spectrometers, which provide universality and specificity. These devices are often combined with chromatographic systems to improve detection capabilities and overcome the above challenges [17]**.** In addition to improving detection capabilities, scientists have focused on refining sample preparation techniques. Conventional methods typically involve hazardous organic solvents, are time-consuming, expensive and require labour-intensive procedures [25,26]. The QuEChERS (Quick, Easy, Cheap, Effective, Rugged, and Safe) approach has achieved global acknowledgment for its simplicity and versatility [27,28]. The QuEChERS method was also produced for application in gas chromatography and liquid chromatography–tandem mass spectrometry (LC–MS/MS) [28,29].

This study aims to validate a QuEChERS (quick, easy, cheap, effective, rugged, and safe) method alongside the use of ultra-performance liquid chromatography and tandem MS (UPLC–MS/MS) to analyse 90 pesticide residues from different classes in three different types of pepper fruits. Subsequently, 536 fresh peppers collected from 2023 in the Al-Qassim region of Saudi Arabia were analysed using the validated method. After detecting and quantifying these residues, we examined the quality of the pepper products using the index of quality for residues (IqR). Additionally, the potential long-term health risks of the detected pesticide residues for adult and child consumers were examined. This comprehensive study provides valuable insights into the pesticide residues associated with green bell, green hot and red chilli peppers in Saudi Arabia.

## 2 Materials and methods

### 2.1 Chemicals and reagents

HPLC-grade methanol, acetonitrile, and LC–MS-grade ammonium formate and formic acid were from Fisher Scientific Ltd. (Loughborough, UK). Anhydrous magnesium sulfate (MgSO_4_), sodium chloride (NaCl), tri-sodium citrate (C_6_H_5_Na_3_O_7_), and di-sodium hydrogen citrate sesquihydrate (C_6_H_6_Na_2_O_71_.5H_2_O) were purchased from Chem-Lab NV (Zedelgem, Belgium). Primary secondary amine (PSA) was purchased from Agilent Technologies Inc. (DE, USA). Multi-walled carbon nanotube (MWCNTs) was purchased from Shilpent Co. (Shilpa Enterprises, Maharashtra, India). Ultra-pure deionised water was produced using an Evoqua Water purification system (Günzburg, Bavaria, Germany). Certified reference pesticide standards (90 pesticides) of 97%–99.5 % purity were purchased from Chem Service Inc. (West Chester, PA, USA).

Certified reference standards for pesticide stock solutions, with a concentration of 1000 mg/l, were each made in acetonitrile and stored in glass volumetric flasks at −20 °C until needed for testing. Working solutions of mixed standards at various concentrations were then produced by diluting specific amounts of these stock solutions in acetonitrile and were preserved at 4 °C in a freezer.

### 2.2 Samples collection

From January to December 2023, 536 ripe pepper fruits were collected from various markets in the Al-Qassim region of Saudi Arabia. This collection included 206 green bell, 180 red chilli and 150 green hot peppers, according to the standardised sampling guidelines issued by the European commission [30]. The weight of each sample collected was at least 2 kg. External substances, such as soil outside the fruit, were removed before further processing. The fruits were used for analysis without being washed or peeled. They were then cut into approximately 2–3-cm pieces and frozen overnight at −20 °C. A Retsch GM-200 food processor blender (Retsch GmbH, Haan, Germany) was used for homogenisation. The homogenised pepper fruit samples were stored in airtight polyethylene containers at −20 °C until they were analysed, which was performed within 1 week following homogenisation.

### 2.3 Extraction and clean-up

Frozen, homogenised pepper sample (10 ± 0.2 g) was weighed into a 50-mL centrifuge tube and then 10-mL acetonitrile was added to it. The mixture was subsequently shaken vigorously for 2 min. A salt mixture of 4-g anhydrous magnesium sulfate (MgSO_4_), 1-g sodium chloride (NaCl), 1-g tri-sodium citrate, and 0.5-g di-sodium hydrogen citrate sesquihydrate was added. The tube was shaken vigorously for 30 s after inserting ceramic piece and centrifuged for 5 min at 5000 rpm using a Universal 320 centrifuge (Hettich, Germany). Subsequently, 2 mL of the supernatant was carefully transferred to a 15-mL conical centrifuge tube containing 300-mg MgSO_4_, 50-mg PSA and 5-mg MWCNTs. This tube was then shaken for 1 min and centrifuged at 5000 rpm for 5 min. The resulting clarified extract was finally filtered through a 22-μm syringe filter into a vial for LC–MS/MS analysis.

### 2.4 LC–MS/MS analysis

For chromatographic separation, a Thermo Scientific™ Dionex Ultimate™ 3000 RS UHPLC + system with an Accucore Q C_18_ analytical column (2.6 μm, 2.1 mm × 150 mm) was used and the temperature was maintained at 40 °C (Thermo Fisher Scientific, Austin, USA). The LC parameters included deionised water as mobile phase A and methanol as mobile phase B containing 0.1 % formic acid and 5-mM ammonium formate. The mobile phase gradient was programmed to maintain 2 % B for the first 1 min, then increased to 35 % from 1 to 5 min, then to 98 % from 5 to 10 min, and maintained at 98 % for 4 min, subsequently returning to 2 % at 14.1 min and maintained for 20 min. The setup included an injection volume of 2 μl and a 0.3 mL/min flow rate. Mass spectrometric analysis was conducted using a TSQ Altis triple quadrupole mass spectrometer (Thermo Fisher Scientific, Austin, USA) in the positive electrospray ionisation mode (H-ESI^+^). The mass spectrometer source conditions included a gas flow of 10 l/min, ion transfer tube and evaporator temperatures of 325 °C and 350 °C, respectively, sheath and auxiliary gas pressures of 40 and 10 Arb, respectively, nebuliser pressure of 40 psi and capillary voltage of 3500 V. The operation of the UHPLC–MS/MS system was controlled using the Trace Finder software (v.4.1). Full details of the compounds analysed, including retention times, MRM transitions for quantification and qualification and the specific collision energies selected, are presented in Table S1.

### 2.5 Method validation

The method performance criteria, including the analytical linearity range, limits of detection (LOD), limit of quantification (LOQ), accuracy, precision, and matrix effect (ME%), were evaluated following SANTE/11312/2021 [31] guidelines.

Linearity was assessed using calibration curves matched to the matrix. Concentrations from 1 to 500 μg/l were analysed in duplicate across eight levels. The criteria for a satisfactory analytical calibration curve included a deviation of the individual residuals of <20 % and a coefficient of determination (R^2^) of at least 0.99. The recovery percentage, which was used to assess the method's accuracy, was determined at three fortification levels (three replicates for each level) by adding specific volumes of mixed pesticide standard solutions to a pepper sample previously tested to confirm the absence of pesticide residues, then extracted and cleaned up as mentioned above.

The precision measured in terms of the intra-day repeatability (RSDr) test was conducted by analysing spiked samples (n = 6) on the same day and inter-day repeatability (RSDR) by analysing spiked samples (n = 18) on three different days with breaks of 7 d. The LOD for the compounds of interest spiked in the final sample extract was determined by identifying the concentrations at which a signal-to-noise ratio (S/N) of 3 was achieved. The method's LOQ corresponds to the minimum enrichment that meets the acceptance criteria for recovery and precision of 70%–120 % and 20 %, respectively. MEs were determined by comparing the analytical slopes when the standards were prepared in pure acetonitrile and the final matrix extract. Two calibration curves were generated to cover the concentration range applicable to all the analytes. The value of the ME% was determined by applying Equation (1):

(1) $$\%\text{ME}=\left(\frac{\text{slope}\;\text{of}\;\text{matrix}-\text{matched}\;\text{calibration}\;\text{curve}}{\text{slope}\;\text{of}\;\text{the}\;\text{solvent}\;\text{calibration}\;\text{curve}}\;-\;1\right)\times 100$$

(Equation (1)) MEs ranging from −20 % to +20 % were considered weak, those ranging from −50 % to −20 % or +20 % to +50 % were considered medium, and those ranging from <−50 % or >+50 % were considered strong [32,33]**.**

### 2.6 Index of quality for residues (IqR)

The *IqR* was used to evaluate the impact of the concentrations of the different pesticides on the overall quality of the pepper samples. It was calculated for each sample by adding the ratio between the pesticide residue content (PRC) in mg/kg and the maximum residue limit (MRL) in mg/kg for each pesticide (Equation (2)). The quality of the samples was categorised into four levels: excellent for *IqR* equal to 0, good for IqR ranging from 0 to 0.6, adequate for *IqR* between 0.6 and 1.0 and inadequate for *IqR* of >1.0 [34]**.**

(2) $$IqR=\sum_{i=1}^{n}PRCi/MRLi$$

i: index of the pesticides found in each contaminated sample.

### 2.7 Health risk estimation

The chronic hazard quotient (HQ_c_) for a given pesticide was calculated by dividing the dietary exposure to that pesticide from the consumption of peppers (Equation (3)) by the ADI for the pesticide (Equation (4)) [35,36]:

(3) $$\text{EDI}=\frac{\text{Mean}\;\text{residue}\;\left(\text{mg}/\text{kg}\right)\times F\left(\text{kg}\right)}{Body\;wieght\;\left(kg\right)}$$

(4) $${\text{HQ}}_{c}=\frac{EDI}{ADI}$$

EDI represents the estimated daily intake, F (in kg/day) denotes the per capita average daily pepper consumption, bw stands for the average body weight (in kg), and ADI is the acceptable daily intake (in mg/kg.bw). The daily average intake of pepper fruits (specifically the subgroup of raw peppers, *Capsicum* spp. only, excluding okra) is 7.63 g/day. Meanwhile, the average body weight is 53 kg for adults and 16 kg for children aged 2–6 years.

Cumulative chronic dietary exposure was examined by determining the hazard index (HI_c_), which is the sum of the chronic hazard quotients (HQ_c_) for all pesticides and was calculated using Equation (5) [37]**.**

(5) HIc=∑HQc

If HQc and HIc values are <1, the health risk is acceptable; however, if all values surpass 1, the risk is deemed unacceptable [38]**.**

## 3 Results and discussions

### 3.1 Validation of the method

#### 3.1.1 Range, linearity, and MEs

The linear range was obtained for 46 of 90 pesticides at 1–100 μg/l, for 27 of 90 pesticides at 5–200 μg/l, for 8 of 90 pesticides at 10–200 μg/l, for 7 of 90 pesticides at 2.5–200 μg/l, for 8 of 90 pesticides at 2.5–100 μg/l, and 1 of 90 pesticides at 10–500 μg/l. The pesticides tested exhibited strong linearity, as evidenced by a coefficient of determination (R^2^) of >0.992 and relative residuals of ≤16.63 % (Table S2).

Slope comparisons of the calibration curves showed MEs classified as weak (signal changes ±20%–0%), moderate (±50%–±20 %), and strong (>±50 %) [39]**.**

In all matrices, 61 of 90 pesticides exhibited signal suppression compared with solvent calibration, whereas 29 of 90 pesticides exhibited signal improvement in all the matrices.

For 15 of 90 pesticides in red chilli peppers and 8 of 90 in green hot peppers, ME showed moderate signal suppression or enhancement, i.e. >20 % or < −20 %. Conversely, a moderate signal suppression or enhancement was detected for 75 pesticides in red chilli, 82 pesticides in green hot peppers, and all 90 pesticides in bell peppers (Table S2). No relation was observed between the retention time and the ME. As MEs cannot be eliminated [40,41], calibration curves matched to each matrix were generated to mitigate quantification inaccuracies caused by MEs.

#### 3.1.2 LOD and LOQ

The LOD was experimentally determined by spiking all analytes with blank pepper extracts. Generally, the LODs determined ranged from 0.0013 to 0.0062 mg/kg.

Table S2 shows the LOQ for each compound. As shown, 47 of 90 pesticides had LOQs of 0.005 mg/kg, with acceptance criteria of recoveries and precisions of 88.9%–93.2 % and ≤13.4 %, respectively. Furthermore, 36 of 90 pesticides had LOQs of 0.01 mg/kg, with acceptance criteria of recoveries and precisions of 92.8%–96.4 % and ≤12.7 %, respectively. Only seven pesticides had LOQs of 0.025 mg/kg, with acceptance criteria for recoveries and precisions of 86.7%–91.7 % and ≤15.8 %, respectively.

#### 3.1.3 Accuracy

**Fig. 1:**
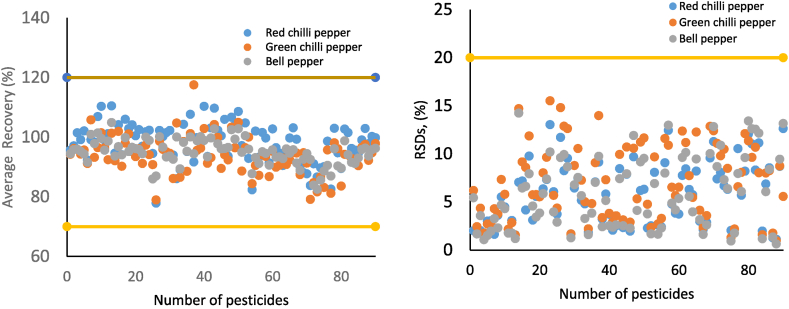
Average recovery (%) and pooled RSDs (%) of the target analytes at 0.01, 0.1, and 1 mg/kg spike levels in bell, green hot, and chili peppers.

Recovery tests were conducted to evaluate the method's accuracy. The recoveries obtained at three different spike concentrations—0.01, 0.1, and 1 mg/kg—are shown in Table S3. For azoxystrobin, bifenazate, buprimate, cyantranilprole, fluopyram, metaflumizone, methoxyfenozide, metrafenone, myclobutanil, and tebufenozide, the tests were conducted at a spike concentration of 3 mg/kg to adhere to the specified MRL (EU-MRL database) [9]. For pesticides with MRLs above the working range, the method's applicability should be assessed via recovery tests with spiked samples above the MRL and subsequent appropriate dilution with the final blank extract at a dilution factor that ensures that the concentration is within the working range. As shown in Fig. 1 and Table S3, the recoveries and RSDs are within the ranges of 76.8%–113.5 % and 0.8%–17.5 %, 76.2%–117 % and 0.3%–13.8 % and 77.1%–114.9 % and 0.4%–19.5 % for spiking levels of 0.01, 0.1 and 1 mg/kg, respectively, and 80.3%–112.8 % and 0.4%–14.4 % for the 10 pesticides spiked at 3 mg/kg. As shown in Fig. 1 and Table S3, all results were within an acceptable 70%–120 % range.

#### 3.1.4 Precision

The method's precision was examined by determining the relative standard deviation for both intra-day and inter-day repeatability (represented as RSDr% and RSD_R_%, respectively) at the levels of the LOQs for the tested pesticides (Fig. 1 and Table S2) The intra-day repeatability (RSDr) ranged from 1 % to 14.8 %, whereas the inter-day repeatability (RSD_R_) ranged from 2.2 % to 19.7 % of the analytes tested in the matrices tested. The results' accuracy and precision were within the ranges recommended by the SANTE/11312/2021 guideline (70%–120 % and <20 %, respectively), showing that the method meets a good standard for analysing the tested pesticides in pepper samples.

### 3.2 Pesticide residue occurrence in pepper fruits

Herein, 536 pepper samples from the Al-Qassim region of Saudi Arabia were collected and analysed for the residues of 90 different pesticides. The detected pesticides and their frequencies are summarised in Table 1, Table 2, Table 3.

**Table 1:** Occurrence of pesticide residues in green bell pepper fruits.

Pesticides	MRL (mg/kg)	Frequency (N, %)	Range (mg/kg)	Mean (mg/kg)	Samples < MRL (N, %)	Samples > MRL (N, %)
Acetamiprid	0.3	38, 18.45	0.023–1.098	0.174	31, 15.05	7, 3.39
Azoxystrobin	3	19, 9.22	0.017–0.608	0.159	19, 9.22	
Bifenazate	3	16, 7.77	0.016–0.594	0.137	16, 7.77	
Buprofezin	0.01	4, 1.947	0.041–0.151	0.080		4, 1.94
Cyflumetofen	0.01	8, 3.89	0.036–0.305	0.130		8, 3.88
Carbendazim	0.1	10, 4.85	0.022–0.083	0.043	10, 4.85	
Clothianidin	0.04	7, 3.39	0.014–0.073	0.051	2, 0.97	5, 2.43
Chlorantranilprole	1	3, 1.46	0.031–0.061	0.042	3.5, 1.69	
Dinotefuran	5	3, 1.46	0.016–0.087	0.042	3, 1.46	
Difenoconazole	0.9	18, 8.74	0.023–0.342	0.091	18, 8.73	
Etoxazole	0.1	9, 4.37	0.023–0.158	0.076	7, 3.39	2, 0.97
Fenhexamid	3	7, 3.39	0.029–0.967	0.413	7, 3.39	
Fenpyroximate	0.3	2, 0.97	0.035–0.117	0.076	2, 0.97	
Fluopyram	2	11, 5.34	0.022–0.381	0.141	11, 5.34	
Bifenthrin	0.5	1, 0.49	0.189	0.189		1, 0.49
Hexythiazox	0.09	6, 2.91	0.037–0.086	0.061	6, 2.91	
Indoxacarb	0.3	19, 9.22	0.026–0.343	0.099	18, 8.74	1, 0.49
Imidacloprid	0.9	60, 29.13	0.031–1.306	0.287	59, 28.64	1, 0.49
Methomyl	0.04	4, 1.94	0.033–0.144	0.085	1, 0.49	3, 1.46
Methoxyfenozide	2	5, 2.43	0.012–0.371	0.111	5, 2.43	
Metalaxyl	0.5	8, 3.88	0.042–0.323	0.126	8, 3.88	
Metaflumizone	1.5	1, 0.49	0.073	0.073	1, 0.49	
Pyriproxyfen	1	3, 1.46	0.032–0.132	0.092	3, 1.46	
Pyraclostrobin	0.5	4, 1.941	0.037–0.101	0.061	4, 1.94	
Propiconazole	0.01	3, 1.46	0.023–0.151	0.071		3, 1.46
Pyridaben	0.3	11, 5.33	0.021–0.235	0.070	11, 5.34	
Spiromesifen	0.5	5, 2.43	0.036–0.264	0.105	5, 2.43	
Spinosad	0.6	2, 0.97	0.035–0.101	0.068	2, 0.97	
Thiophanate-methyl	0.1	22, 10.68	0.024–0.257	0.105	11, 5.34	11, 5.34
Tebuconazole	0.6	2, 0.97	0.049–0.093	0.071	2, 0.97	
Thiamethoxam	0.7	20, 9.7	0.015–0.553	0.119	20, 9.71	
Trifloxystrobin	0.4	9, 4.37	0.036–0.428	0.152	8, 3.88	1, 0.49

**Table 2:** Occurrence of pesticide residues in green hot pepper fruits.

Pesticides	MRL (mg/kg)	Frequency (N, %)	Range (mg/kg)	Mean (mg/kg)	Samples < MRL (N, %)	Samples > MRL (N, %)
Acetamiprid	0.3	19, 12.67	0.031–0.664	0.216	14, 9.33	5, 3.33
Azoxystrobin	3	13, 8.67	0.027–0.887	0.256	13, 8.67	
Bifenazate	3	11, 7.33	0.024–0.234	0.090	11, 7.33	
Buprofezin	0.01	4, 2.67	0.075–0.169	0.110		4, 2.67
Carbendazim	0.1	12, 8	0.041–0.999	0.177	6, 4	6, 4
Chlorantranilprole	1	3, 2	0.052–0.115	0.074	3, 2	
Cyflumetofen	0.01	4, 2.67	0.011–0.276	0.130		4, 2.67
Clothianidin	0.04	6, 4	0.041–0.191	0.101		6, 4
Difenoconazole	0.9	15, 10	0.031–1.224	0.253	13, 8.67	2, 1.33
Fenpyroximate	0.01	1, 0.67	0.126	0.126		1, 0.67
Fipronil	0.005	2, 1.33	0.135–0.286	0.211		2, 1.33
Bifenthrin	0.5	1, 0.67	0.094	0.094	1, 0.67	
Fluopyram	2	2, 1.33	0.053–0.132	0.093	2, 1.33	
Hexythiazo	0.09	2, 1.33	0.032–0.053	0.043	2, 1.33	
Imidacloprid	0.9	35, 23.33	0.019–0.852	0.193	35, 23.33	
Indoxacarb	0.3	10, 6.67	0.023–0.094	0.056	10, 6.67	
Myclobutanil	3	1, 0.67	0.129	0.129	1, 0.67	
Metaflumizone	1.5	1, 0.67	0.069	0.069	1, 0.67	
Metalaxyl	0.5	5, 3.33	0.048–0.911	0.272	4, 2.67	1, 0.67
Pyraclostrobin	0.5	7, 4.67	0.023–0.353	0.214	7, 4.67	
Pyridaben	0.3	10, 6.67	0.025–1.074	0.321	7, 4.67	3, 2
Propiconazole	0.01	1, 0.67	0.045	0.045		1, 0.67
Spiromesifen	0.5	2, 1.33	0.072–0.121	0.097	2, 1.33	
Thiophanate-methyl	0.1	16, 10.67	0.051–0.559	0.302	3, 2	13, 8.67
Tebuconazole	0.6	6, 4	0.029–0.218	0.108	6, 4	
Thiamethoxam	0.7	6, 4	0.027–0.256	0.144	6, 4	
Trifloxystrobin	0.4	3, 2	0.021–0.518	0.187	2, 1.33	1, 0.67

**Table 3:** Occurrence of pesticide residues in red chili pepper fruits.

	MRL (mg/kg)	Frequency (N, %)	Range (mg/kg)	Mean (mg/kg)	Samples < MRL (N, %)	Samples > MRL (N, %)
Azoxystrobin	3	17, 9.44	0.025–0.164	0.067	17, 9.44	
Acetamiprid	0.3	37, 20.56	0.014–1.368	0.239	29, 16.11	8, 4.44
Bifenazate	3	39, 21.67	0.022–1.808	0.133	39, 21.67	,
Buprofezin	0.01	9, 5	0.022–0.508	0.274		9, 5
Bifenthrin	3	1, 0.56	0.102	0.102	1, 0.56	,
Carbendazim	0.1	4, 2.22	0.031–0.151	0.070	3, 1.67	1, 0.56
Clothianidin	0.04	10, 5.56	0.021–0.127	0.051	5, 2.78	5, 2.78
Chlorantranilprole	1	5, 2.78	0.023–0.075	0.045	5, 2.78	,
Cyantranilprole	1.5	3, 1.67	0.032–0.061	0.048	3, 1.67	,
Cyflumetofen	0.01	18, 10	0.018–0.451	0.193		18, 10
Difenoconazole	0.9	33, 18.33	0.014–0.746	0.150	33, 18.33	,
Dinotefuran		9, 5	0.022–0.225	0.129	9, 5	,
Etoxazole	0.1	3, 1.67	0.027–0.121	0.075	2, 1.11	1, 0.56
Fluopyram	2	13, 7.22	0.018–0.471	0.106	13, 7.22	,
Fipronil	0.005	1, 0.56	0.013	0.013		1, 0.56
Fenpyroximate	0.3	6, 3.33	0.022–0.108	0.052	6, 3.33	,
Hexythiazox	0.09	9, 5	0.026–0.138	0.054	8, 4.44	1, 0.56
Indoxacarb	0.3	14, 7.78	0.021–0.219	0.054	14, 7.78	,
Imidacloprid	0.9	76, 42.22	0.017–0.983	0.265	75, 41.67	1, 0.56
Metalaxyl	0.5	8, 4.44	0.023–0.164	0.064	8, 4.44	,
Myclobutanil	3	3, 1.67	0.051–0.179	0.095	3, 1.67	,
Methoxyfenozide	2	3, 1.67	0.018–0.118	0.057	3, 1.67	,
Pyraclostrobin	0.5	9, 5	0.029–0.299	0.094	9, 5	,
Pyriproxyfen	1	5, 2.78	0.022–0.318	0.093	5, 2.78	,
pyridaben	0.3	23, 12.78	0.021–0.621	0.121	20, 11.11	3, 1.67
Propiconazole	0.01	7, 3.89	0.022–0.551	0.142		7, 3.89
Spinosad	0.6	2, 1.11	0.016–0.033	0.025	2, 1.11	,
Spiromesifen	0.5	9, 5	0.028–0.306	0.137	9, 5	,
Tebuconazole	0.6	4, 2.22	0.045–0.224	0.109	4, 2.22	,
Trifloxystrobin	0.4	6, 3.33	0.025–0.556	0.177	5, 2.78	1, 0.56
Thiophanate-methyl	0.1	13, 7.22	0.028–0.325	0.120	7, 3.89	6, 3.33
Thiamethoxam	0.7	20, 11.11	0.021–0.445	0.118	20, 11.11	

#### 3.2.1 Green bell pepper

A total of 206 samples of green bell peppers were analysed in this study (Table 1). Of these samples, 64 (31.07 %) were free of any detectable pesticides. While 142 (68.93 %) of the samples were contaminated with 32 different pesticides, 31 (15.05 %) samples were contaminated with a single pesticide residue, and 111 (53.88 %) samples were contaminated with multiple pesticides. Of the 32 pesticides, 12 were detected to have exceeded the MRLs. Additionally, 37 (17.96 %), 6 (2.61 %) and 1 (0.49 %) of the 206 samples were contaminated with 1, 2 and 3 pesticides, respectively, that exceeded their MRLs.

The residues of the neonicotinoids imidacloprid and acetamiprid were detected in 29.13 % (60 samples) and 18.45 % (38 samples) of the tested samples, respectively. The detected concentration ranges were 0.031–1.01 mg/kg for imidacloprid and 0.023–1.09 mg/kg for acetamiprid. Additionally, other pesticides’ residue, namely, thiophanate methyl, thiamethoxam, indoxacarb, azoxystrobin, difenoconazole, bifenazate (including bifenazate diazene) and fluopyram, were detected at varying frequencies: 10.68 % (22 samples) for thiophanate methyl, 9.71 % (20 samples) for thiamethoxam, 9.22 % (19 samples) for indoxacarb, 8.74 % (18 samples) for azoxystrobin, 7.37 % (16 samples) for difenoconazole and 5.37 % (11 samples) for fluopyram.

Twenty-three pesticide residues were detected with a frequency of <5 %. Significantly, half of the samples (11 of 22 samples) contaminated with thiophanate methyl residues at concentrations between 0.102 and 0.257 mg/kg exceeded the MRL of 0.1 mg/kg. Furthermore, 8 samples contaminated with cyflumetofen exceeded the MRL of 0.01 mg/kg and showed concentrations between 0.036 and 0.305 mg/kg. Clothianidin was detected in seven samples (3.39 %) at concentrations of 0.014–0.073 mg/kg, with five samples exceeding the MRL of 0.04 mg/kg. Buprofezin residues were found in 4 samples, accounting for 1.95 % of the total, with detected concentrations ranging from 0.041 to 0.151 mg/kg, above the MRL set at 0.01 mg/kg. The residues of propiconazole and methomyl were detected in three samples, exceeding the MRLs of 0.01 mg/kg for propiconazole and 0.04 mg/kg for methomyl, with concentrations varying between 0.023 and 0.151 mg/kg for propiconazole and 0.057–0.144 mg/kg for methomyl.

#### 3.2.2 Green hot peppers

The analysis of the pesticide residues of 150 samples of green hot peppers revealed that 92 samples (61.34 %) contained residues of 27 different pesticides. More specifically, 37 samples (24.66 %) contained residues of a single pesticide, whereas 55 samples (36.66 %) contained multiple pesticide residues (Table 2). The neonicotinoids imidacloprid and acetamiprid were detected most frequently, at a detection rate of 23.33 % and 12.67 %, respectively. Notably, 3.33 % of the samples contaminated with acetamiprid exceeded the MRL of 0.3 mg/kg, whereas none of the samples contaminated with imidacloprid exceeded the MRL of 0.9 mg/kg. The fungicides thiophanate methyl and difenoconazole were found at 0.051–0.559 mg/kg and 0.031–1.224 mg/kg, respectively, and were present in ∼10 % of the contaminated samples. Furthermore, 8.67 % and 1.33 % of the samples exceeded their respective MRLs of 0.9 and 0.1 mg/kg. Azoxystrobin, bifenazate, and indoxacarb were detected at different concentrations in the samples; however, none of the concentrations exceeded their respective MRLs of 3, 3, or 0.3 mg/kg. However, carbendazim and pyridaben were present in 8 % and 6.67 % of the samples and exhibited concentrations between 0.041 and 0.999 mg/kg and between 0.024 and 1.074 mg/kg, respectively. Of these, 4 % for carbendazim and 2 % for pyridaben exceeded the MRLs set at 0.1 and 0.3 mg/kg, respectively. Clothianidin, buprofezin and cyflumetofen were identified in 4 %, 2.67 % and 2.67 % of samples, respectively, all of which exceeded their MRLs of 0.04, 0.01 and 0.01 mg/kg, respectively. Metalaxyl and trifloxystrobin were detected in the range of 0.049–0.91 and 0.021–0.51 mg/kg, with a single sample exceeding the MRLs of 0.5 and 0.4 mg/kg, respectively.

#### 3.2.3 Red chilli peppers

In total, 180 samples of red chilli peppers were analysed (Table 3). Of that, 20 samples (11.11 %) were free of the tested pesticides, whereas 160 samples (88.89 %) were contaminated with 32 pesticides. Of these contaminated samples, 23 (12.78 %) contained residues of a single pesticide, 68 (37.78 %) contained residues of two pesticides, and 69 (38.33 %) were contaminated with residues of >2 pesticides. Particularly, the residues of one pesticide were found in 35 samples (19.44 %), residues of 2 pesticides were found in 11 samples (6.11 %), and residues of 3 residues were found in pesticides exceeding their MRLs in 2 samples (1.11 %). Imidacloprid was most frequently detected in 76 samples (42.22 %) with concentrations between 0.017 and 0.983 mg/kg, followed by bifenazate, which was present in 39 samples (21.67 %), with concentrations between 0.022 and 1.808 mg/kg and difenoconazole, which was detected in 33 samples (18.33 %), with concentrations between 0.014 and 0.746 mg/kg. Only one sample contaminated with imidacloprid exceeded the MRL of 0.9 mg/kg.

Acetamiprid was found in 37 samples (20.56 %) at concentrations between 0.014 and 1.368 mg/kg, of which 8 samples (4.44 %) exceeded the maximum residue level of 0.3 mg/kg. Pyridaben was detected in 23 samples (12.78 %) with concentrations between 0.021 and 0.621 mg/kg, and 3 of these samples (1.66 %) exceeded the maximum residue level of 0.3 mg/kg. Thiamethoxam and azoxystrobin appeared in 20 (11.11 %) and 17 (9.44 %) samples, respectively, with thiamethoxam concentrations between 0.021 and 0.445 mg/kg and azoxystrobin concentrations between 0.025 and 0.164 mg/kg, both within their respective MRLs of 0.7 mg/kg for thiamethoxam and 3 mg/kg for azoxystrobin. Alternatively, Cyflumetofen was detected in 18 samples (10 %) at concentrations between 0.018 and 0.451 mg/kg, exceeding the MRL of 0.01 mg/kg.

Additionally, indoxacarb, fluopyram and thiophanate methyl were detected in 14 (7.78 %), 13 (7.22 %), and 13 (7.22 %) samples at concentrations of 0.021–0.219, 0.018–0.471 and 0.105–0.325 mg/kg, respectively. One sample exceeded the specified maximum residue levels for each of these pesticides. Clothianidin was detected in 10 samples (5.56 %) at a concentration range of 0.02–0.127 mg/kg, of which 5 samples (2.77 %) exceeded the maximum residue level of 0.04 mg/kg. Buprofezin was detected in 9 samples (5 %) with concentrations between 0.022 and 0.508 mg/kg, exceeding the maximum residue level of 0.01 mg/kg. Elevated concentrations of carbendazim, etoxazole, fipronil, hexythiazox and trifloxystrobin were also detected in a few individual samples.

The entire data set analysis, which comprised 536 pepper samples, showed that 142 samples (26.49 %) contained no pesticide residues. Of the remaining samples, 394 (73.51 %) contained between 1 and 6 compounds of the detected pesticides, including government-registered and unregistered/banned substances. The 36 identified pesticides were categorised into 14 insecticides (38.9 %), 14 fungicides (38.9 %) and 8 acaricides (22.2 %). Notably, the overall detection rate of pesticides in red chilli peppers (232 %) was significantly higher than in bell peppers (165 %) and green chilli peppers (132 %), with the highest residue concentrations observed in red chilli peppers. Neonicotinoids and triazoles stood out in this study as the most frequently detected pesticides. They are characterised by their extensive target spectrum, high efficiency and favourable ecological and biological properties. These chemicals are mainly used in Saudi Arabia instead of organophosphates to increase the quality and productivity of pepper cultivation [[42], [43], [44]].

**Fig. 2:**
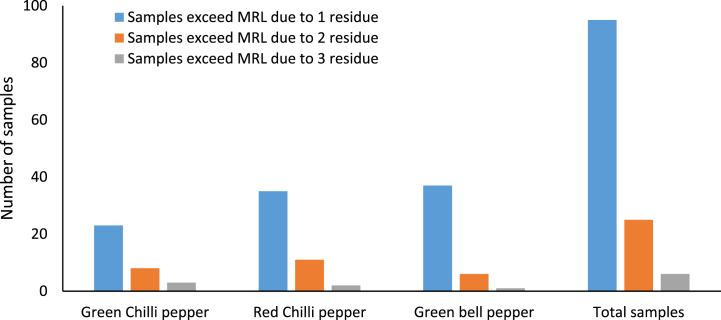
The distribution of pepper fruits exceeded the MRL.

Additionally, pesticides of the Strobulin and hydrazide classes were frequently detected, accounting for 7 % and 6.9 % of the detections, respectively, whereas 10.34 % were categorised into unclassified groups. Pesticide residues exceeded the MRLs in 126 samples (23.51 %). Particularly, 17.72 % of the samples exceeded the MRLs owing to contamination with a single residue, followed by 4.66 % and 1.12 % of the samples contaminated with two and three residues, respectively (Fig. 2). Imidacloprid proved to be the most frequently detected pesticide and was present in 171 samples (31.9 %), followed by acetamiprid (94 samples, 17.54 %), bifenazate (66 samples, 12.31 %) and difenoconazole (66 samples, 12.31 %). Among the other pesticides, 24 were found in 1%–10 % of the samples and 8 were present in <1 %.

The increased concentrations of insecticides and fungicides are due to their extensive use as preservatives during harvest. Pests such as aphids, whiteflies and mites, which feed on the leaves and stems of pepper plants, and fungal diseases such as anthracnose and blossom end rot, which are characterised by leaf and fruit discoloration, wilting and tissue necrosis, necessitate the use of these chemical agents [16]. Therefore, farmers often resort to insecticides and fungicides to increase the yield of peppers by containing pest infestations and thus improving overall profitability [45]**.** The non-detection of organophosphorus pesticides in the examined samples signifies their restriction in Saudi Arabia. Particularly, insecticide residues predominate over fungicide residues, indicating that pepper plantations are more susceptible to pest infestation than disease. Furthermore, three banned pesticides—thiophanate methyl, carbendazim and methomyl—were detected in 9.07 %, 3.63 % and 1.04 % of the samples, respectively, in our study. However, the banned insecticide, fipronil, was found in 0.26 % of the samples.

Carbendazim detection, especially with its precursor, thiophanate methyl, underlines the urgent need to include metabolites in routine monitoring [46]**.** This finding suggests that MRLs for thiophanate methyl and carbendazim should be re-evaluated and advocates stricter regulations for banned pesticides. It also recommends reducing commonly used pesticides or replacing them with safer alternatives. Exceeding MRLs and the detection of banned or restricted pesticides indicate deviations from good agricultural practice [47]**.**

Yıldırım and Çiftçi analysed 108 bell pepper samples from Turkish markets in 2022, testing for 283 pesticide residues. The examination found pesticide residues in 68.5 % (74 samples) and no residues in 31.5 % (34 samples) of the samples. Furthermore, 36.5 % of the contaminated samples contained one pesticide residue, and 63.5 % contained several residues, with 3 samples containing 6–8 residues. The detailed data showed that 5 samples (4.6 %) contained 5 residues, 10 (9.2 %) contained 4 residues, 14 (13.9 %) contained 3 residues, and 15 (13.9 %) contained 2 residues [17]**.** Ersoy et al. (2011) examined pesticide residues in Konya market and bazaar vegetable samples. They found prohibited pesticides such as ethion, triazophos and benomyl-carbendazim at amounts above the MRLs [19]**.** In contrast to our results, Kaya and Tuna (2019) analysed pesticide residues in a modest collection of 42 fruit and vegetable samples from three different bazaars in İzmir province. They found no residues exceeding the MRLs in peppers [48]**.** Similarly, Sungur and Tunur (2012) analysed 175 pesticide residues in fruits and vegetables in Hatay province and found that none of the pepper samples (10 samples) exceeded the MRLs [49]**.** Further investigations in Turkey with 325 samples of peppers from markets and bazaars in Adana, Mersin and Antalya showed that although 13.2 % (96 samples) contained detectable pesticide residues, none exceeded the EU MRLs [50]**.**

### 3.3 Multi-residues and quality assessment of pepper fruits

**Fig. 3:**
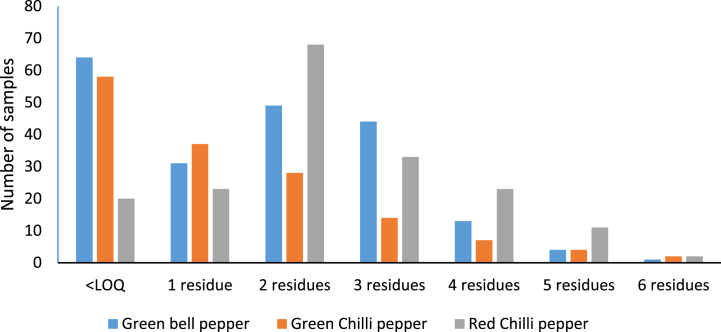
Distribution of multiple pesticide residues in individual pepper samples.

The presence of several pesticide residues in a sample often indicates that the plants have been exposed to different pesticide applications. Such residues can originate from the soil, especially in the case of pesticides that remain in the environment. Additionally, spray drift from neighbouring fields or cross-contamination during crop processing can contribute to residue levels [51]**.** The distribution of pesticide contamination per sample was as follows: 91 samples (16.98 %) with 1 residue, 145 samples (27.05 %) with 2 residues, and a decreasing frequency for samples with 3–6 residues (Fig. 3). Particularly, green hot peppers were more likely to contain multiple residues than green bell and red chilli peppers. Up to six different pesticides were detected in a single sample of green bell peppers, whereas the samples of green hot peppers and red chilli peppers contained mainly two pesticides. Red chilli peppers (12.69 %), green bell pepper (9.14 %), and green hot peppers (5.22 %) exhibited the highest prevalence of samples with duplicate pesticide residues, with a decreasing trend as the number of residues increased (Fig. 3).

International studies underline this phenomenon: in Shanxi Province, China, 6 % of samples had two or more pesticides [52]**,** compared with 8 % in Poland [53], 33.3 % in Thailand [54] and 20 % in Turkey [55]**.** Ghana reported a significantly higher rate, with 70.7 % of vegetable samples containing multiple residues [56]. In Saudi Arabia, single residues were found in 12.8 % of pepper samples, whereas 55.4 % contained various residues [20]**.** A similar study in Shandong Province, China, reported that 21.4 % of pepper samples contained single residues and 7.3 % contained multiple residues [21]**.**

Globally, the evaluation of the quality and safety of agricultural products in terms of pesticide residues relies on MRLs. Multiple residues can affect product quality owing to possible additive or synergistic effects. The interquartile range (IqR) is a critical food quality measure [57]**.** It differentiates between low (‘inadequate’) and acceptable (‘good/adequate’) quality. It detects ‘poor quality’ products with residues below the MRL but poses a risk owing to combined pesticide effects.

Herein, the quality assessment of the samples was generally considered satisfactory. Specifically, the samples were categorised as follows: 26.49 % were classified as excellent (IqR = 0), 31.72 % as good (IqR between 0 and 0.6) and 13.06 % as adequate (IqR between 0.6 and 1.0), whereas 28.73 % were classified as inadequate (IqR >1.0; Table 4). The quality of the peppers was rated as excellent (with green hot pepper ahead of green bell pepper and red chilli pepper), good (with red chilli pepper ahead of green bell pepper and green hot pepper), adequate (with red chilli pepper ahead of green bell pepper and green hot pepper) and inadequate (with green bell pepper ahead of green hot pepper and red chilli pepper). These results underline the superior quality and safety of peppers from Saudi Arabia but also emphasise the importance of considering the cumulative risks of pesticide residues, even if they are below the MRLs, as they significantly impact the *IqR*.

**Table 4:** Quality evaluation of the analysed pepper samples according to the calculated IqR factor.

*IqR*^a^	Quality Categories	Green hot pepper		Red Chili pepper		Green bell pepper		Total samples	
		No	%	No	%	No	%	No	%
0	Excellent	58	38.67	20	11.11	64	31.07	142	26.49
0–0.6	Good	41	27.33	72	40.00	57	27.67	170	31.72
0.6–1	Adequate	8	5.33	40	22.22	22	10.68	70	13.06
>1.0	inadequate	43	28.67	48	26.67	63	30.58	154	28.73

a *IqR is the* ratio between the pesticide residue content (PRC) in mg/kg and the maximum residue limit (MRL) in mg/kg for each pesticide.

### 3.4 Chronic and accumulative health risk assessment

The assessment of exposure to pesticide residues included 39 residues. To avoid overestimating the EDI, only residues detected above the LOQ were included in the exposure calculations. Table 5 shows the calculated estimated average daily intake (EDI mg/kg body weight), hazard quotient (HQ), and cumulative chronic hazard index (HIc) for the pesticide residues detected in the pepper samples. The results show that the long-term risk of pesticide exposure from consuming pepper fruits is negligible. Fipronil exhibited the highest HQ of 0.502 for children and 0.152 for the adult population, corresponding to 84 % of the total hazard quotients of green hot and red chilli peppers.

**Table 5:** Estimated dietary exposure, hazard quotient (HQ), and health hazard index for pesticide residues in pepper fruits.

Pesticides	ADI (mg/kg)	Exposure (mg/kg.bw)						HQ					
		Adult			Children			Adult			Children		
		P1	P2	P3	P1	P2	P3	P1	P2	P3	P1	P2	P3
Acetamiprid	0.025	3.12E-05	2.5E-05	3.44E-05	1.03E-04	8.29E-05	1.14E-04	1.25E-03	1.00E-03	1.37E-03	4.13E-03	3.32E-03	4.55E-03
Azoxystrobin	0.2	3.68E-05	2.29E-05	9.65E-06	1.22E-04	7.6E-05	3.2E-05	1.84E-04	1.15E-04	4.83E-05	6.10E-04	3.80E-04	1.60E-04
Bifenazate	0.01	1.29E-05	1.97E-05	1.91E-05	4.28E-05	6.52E-05	6.34E-05	1.29E-03	1.97E-03	1.91E-03	4.28E-03	6.52E-03	6.34E-03
Buprofezin	0.01	1.59E-05	1.14E-05	3.94E-05	5.26E-05	3.79E-05	1.31E-04	1.59E-03	1.14E-03	3.94E-03	5.26E-03	3.79E-03	1.31E-02
Bifenthrin	0.015	–	–	1.47E-05	–	–	4.86E-05	–	–	9.79E-04	–	–	3.24E-03
Cyflumetofen	0.17	1.87E-05	1.87E-05	2.78E-05	6.2E-05	6.19E-05	9.2E-05	1.10E-04	1.10E-04	1.63E-04	3.65E-04	3.64E-04	5.41E-04
Carbendazim	0.02	2.55E-05	6.22E-06	1.01E-05	8.43E-05	2.06E-05	3.34E-05	1.27E-03	3.11E-04	5.04E-04	4.22E-03	1.03E-03	1.67E-03
Clothianidin	0.097	1.45E-05	7.28E-06	7.37E-06	4.81E-05	2.41E-05	2.44E-05	1.50E-04	7.51E-05	7.60E-05	4.96E-04	2.49E-04	2.52E-04
Chlorantranilprole	0.56	1.07E-05	6.09E-06	6.51E-06	3.54E-05	2.02E-05	2.16E-05	1.91E-05	1.09E-05	1.16E-05	6.33E-05	3.60E-05	3.85E-05
Cyantranilprole	0.01	–	–	6.91E-06	–	–	2.29E-05	–		6.91E-04	–	–	2.29E-03
Dinotefuran	0.2		6E-06	1.86E-05	–	1.99E-05	6.16E-05	–	3.00E-05	9.29E-05	–	9.93E-05	3.08E-04
Difenoconazole	0.01	3.64E-05	1.3E-05	2.16E-05	1.30E-04	4.32E-05	7.16E-05	3.64E-03	1.30E-03	2.16E-03	1.20E-02	4.32E-03	7.16E-03
Etoxazole	0.04	–	1.09E-05	1.07E-05	–	3.62E-05	3.56E-05	–	2.73E-04	2.69E-04	–	9.05E-04	8.90E-04
Fenhexamid	0.2	–	5.94E-05	–	–	1.97E-04	–	–	2.97E-04	–	–	9.84E-04	–
Fenpyroximate	0.01	1.81E-05	1.09E-05	7.51E-06	6.01E-05	3.62E-05	2.49E-05	1.81E-03	1.09E-03	7.51E-04	6.01E-03	3.62E-03	2.49E-03
Fipronil	0.0002	3.03E-05	–	1.87E-06	1.00E-04		6.2E-06	1.52E-01		9.36E-03	5.02E-01		3.10E-02
Fluopyram	0.12	1.33E-05	2.03E-05	1.53E-05	4.41E-05	6.73E-05	5.06E-05	1.11E-04	1.69E-04	1.27E-04	3.68E-04	5.61E-04	4.22E-04
Famoxadone	0.006	1.35E-05	2.72E-05		4.48E-05	9.01E-05		2.26E-03	4.53E-03	–	7.47E-03	1.50E-02	
Hexythiazox	0.03	6.12E-06	8.78E-06	7.82E-06	2.03E-05	2.91E-05	2.59E-05	2.04E-04	2.93E-04	2.61E-04	6.76E-04	9.70E-04	8.64E-04
Indoxacarb	0.005	8.08E-06	1.42E-05	7.76E-06	2.68E-05	4.71E-05	2.57E-05	1.62E-03	2.85E-03	1.55E-03	5.35E-03	9.43E-03	5.14E-03
Imidacloprid	0.06	2.78E-05	4.13E-05	3.82E-05	9.2E-05	1.37E-04	1.27E-04	4.63E-04	6.88E-04	6.37E-04	1.53E-03	2.28E-03	2.11E-03
Methomyl	0.0025	–	1.23E-05	–	–	4.07E-05	–	–	4.91E-03	–	–	1.63E-02	–
Metalaxyl	0.08	3.92E-05	1.82E-05	9.27E-06	1.20E-04	6.01E-05	3.07E-05	4.90E-04	2.27E-04	1.16E-04	1.62E-03	7.52E-04	3.84E-04
Metaflumizone	0.01	9.93E-06	1.05E-05		3.29E-05	3.48E-05	–	9.93E-04	1.05E-03	–	3.29E-03	3.48E-03	–
Methoxyfenozide	0.1	–	1.6E-05	8.25E-06	–	5.3E-05	2.73E-05	–	1.60E-04	8.25E-05	–	5.30E-04	2.73E-04
Myclobutanil	0.025	1.86E-05	–	1.37E-05	6.15E-05	–	4.53E-05	7.43E-04		5.47E-04	2.46E-03	–	1.81E-03
Pyraclostrobin	0.03	3.08E-05	8.82E-06	1.36E-05	1.02E-04	2.92E-05	4.5E-05	1.03E-03	2.94E-04	4.53E-04	3.40E-03	9.74E-04	1.50E-03
Pyriproxyfen	0.05	–	1.33E-05	1.34E-05	–	4.4E-05	4.45E-05	–	2.66E-04	2.69E-04	–	8.81E-04	8.91E-04
Pyridaben	0.01	4.63E-05	1.01E-05	1.75E-05	1.53E-04	3.35E-05	5.78E-05	4.63E-03	1.01E-03	1.75E-03	1.53E-02	3.35E-03	5.78E-03
Propiconazole	0.04	6.48E-06	1.02E-05	2.05E-05	2.15E-05	3.37E-05	6.79E-05	1.62E-04	2.54E-04	5.13E-04	5.36E-04	8.42E-04	1.70E-03
Spinosad	0.024	–	9.79E-06	3.53E-06	–	3.24E-05	1.17E-05	–	4.08E-04	1.47E-04	–	1.35E-03	4.87E-04
Spiromesifen	0.03	1.39E-05	1.52E-05	1.97E-05	4.6E-05	5.03E-05	6.53E-05	4.63E-04	5.06E-04	6.57E-04	1.53E-03	1.68E-03	2.18E-03
Tebuconazole	0.03	1.55E-05	1.02E-05	1.57E-05	5.15E-05	3.39E-05	5.21E-05	5.18E-04	3.41E-04	5.24E-04	1.72E-03	1.13E-03	1.74E-03
Thiophanate	0.02	4.35E-05	1.52E-05	1.72E-05	1.44E-04	5.02E-05	5.7E-05	2.17E-03	7.58E-04	8.61E-04	7.20E-03	2.51E-03	2.85E-03
Thiamethoxam	0.026	2.07E-05	1.71E-05	1.69E-05	6.84E-05	5.68E-05	5.61E-05	7.95E-04	6.59E-04	6.51E-04	2.63E-03	2.18E-03	2.16E-03
Trifloxystrobin	0.1	2.7E-05	2.18E-05	2.54E-05	8.93E-05	7.23E-05	8.42E-05	2.70E-04	2.18E-04	2.54E-04	8.93E-04	7.23E-04	8.42E-04
Hic								0.180	0.027	0.032	0.595	0.091	0.105

ADI = acceptable daily intake obtained from European Union Regulations. HQ = hazard quotient. HI = hazard index. P1 = intake for green hot pepper, P2 = intake for bell pepper, and P3 = intake for red chili pepper.

The comprehensive chronic risk assessment revealed that the hazard index (HI) values for pesticide residues in green hot peppers, bell peppers and red chilli were 0.18, 0.027 and 0.031 for adults and 0.595, 0.091 and 0.105 for children aged 2–6 years, respectively, all at a threshold of <1. The level of exposure to each pesticide was well below the ADI established by the European Food Safety Authority [18], with HQs well below 1 for all the pesticides detected (Table 5). Of the 36 pesticides identified, four azoles (difenoconazole, tebuconazole, myclobutanil and propiconazole) contributed the most to the HI, with a share of 2.8 %. Parallel results in Poland and Brazil have also shown that the consumption of fruit poses no health risk for either adults or children [53]**.** By contrast, a study in India has demonstrated that chronic health risks associated with organophosphorus pesticides pose a risk to children [38]. As the human body can accumulate chemical contaminants through food consumption, potentially leading to health problems, the risk of dietary exposure to pesticide residues should be assessed, especially to those considered high-risk [58]**.**

This study's findings argue for stricter regulations and penalties for banned pesticides such as fipronil, thiophanate methyl and methomyl. Introducing more stringent control measures and setting lower thresholds or a complete ban on these high-risk pesticides, especially fipronil, thiophanate methyl and methomyl, is crucial. Uncertainties in assessing dietary risks typically stem from inadequate toxicological or consumption information, processing factors (PFs), handling of data below the detection threshold and the models used for exposure evaluation [59]**.** Household methods such as washing, soaking, peeling and blanching can significantly reduce pesticide residues in fruits and vegetables [60]**.** Additionally, adjusting dietary pesticide exposure with PFs for water, mechanical and thermal processing has shown a 3%–11.5 % reduction in residues [61]**.** However, PFs were not included in the dietary risk assessment in this study, leading to an overestimation of exposure levels. Future studies should aim for a more detailed and realistic assessment of exposure.

## 4 Conclusion

Conclusively, our study reveals the Al-Qassim region's critical role as a pivotal agricultural hub in Saudi Arabia, contributing significantly to the national output of vegetables and fruits. However, lapses in adhering to GAP have been identified, showing the presence of pesticide residues in pepper samples, which could pose potential health risks to consumers. The standard EN-QuEChERS procedure and LC–MS/MS analysis were validated for determining 90 multi-pesticide residues in various pepper types. The method demonstrated high accuracy and precision and was applied to analyse 536 pepper samples collected from the Al-Qassim region, Saudi Arabia, in 2023. Imidacloprid, acetamiprid, bifenazate and difenoconazole were the most frequently detected pesticides. Our findings highlight a concerning level of contamination, particularly in red chilli peppers, which bore the highest pesticide residue concentrations. The more frequent detection of insecticides than fungicides could be because pest infestation by insects occurs more frequently than by pathogens. The IqR is a novel method for classifying the analysed samples. Herein, the analysed pepper fruits ranged from excellent to adequate. However, a substantial fraction fell into the inadequate category. This classification system provided insightful evaluations regarding pepper fruit quality, revealing a nuanced understanding of pesticide residue distribution across different pepper types. By leveraging food consumption data specific to Saudi Arabia, we also examined the potential health risks to consumers. Our results suggest that the pesticide residues found in peppers do not pose a risk to consumers in Saudi Arabia. The study also emphasises the importance of ongoing monitoring to safeguard public health and facilitate safe trade practices in addition to highlighting the urgent need for regulatory oversight in the agricultural sector of the Al-Qassim region and providing a comprehensive analytical framework for monitoring and managing pesticide residues in food crops, thereby contributing to the global endeavour of ensuring food safety.

## Supplementary materials

Table S1. UPLC-MS/MS parameters for determining pesticides in pepper, with retention time, RF lens, precursor and product ions, and collision energy for both transitions monitored. Table S2. Linearity range, limit of quantitation (LOQ), intra-day (RSDr), inter-day (RSD_R_) repeatability, matrix effect (%ME), and EU-MRL of the tested pesticides in pepper fruits. Table S3. Percentage recoveries and relative standard deviation (RSD) of the tested pesticides in pepper fruits.

## Data availability statement

Data is included in the article/supp. material/referenced in the article.

## CRediT authorship contribution statement

**Fahad M. Alminderej:** Writing – review & editing, Resources, Funding acquisition, Data curation. **Sayed M. Saleh:** Writing – original draft, Validation, Formal analysis, Data curation. **Osama I. Abdallah:** Writing – original draft, Validation, Methodology, Formal analysis, Data curation, Conceptualization.

## Declaration of competing interest

The authors declare that they have no known competing financial interests or personal relationships that could have appeared to influence the work reported in this paper.
