# Supplementary material for: Monitoring pesticide residues in pepper (Capsicum annuum L.) from Al-Qassim region, Saudi Arabia: Occurrence, quality, and risk evaluations
Source: Heliyon. 2024 Aug 29;10(17):e36805. doi: 10.1016/j.heliyon.2024.e36805 (PMC11408781; doi:10.1016/j.heliyon.2024.e36805)
Supplement: Multimedia component 1 [file mmc1.docx]

Supporting information

**Monitoring Pesticide Residues in Peppers (*Capsicum annuum* L.) from Al-Qassim region, Saudi Arabia: Occurrence, Quality, and Risk Evaluations**

Table S1. UPLC-MS/MS parameters for determining pesticides in pepper, with retention time, RF lens, precursor and product ions, and collision energy for both transitions monitored.

| Pesticides | Retention Time (min) | Precursor (m/z) | Quantifier ion (m/z) | CE (V) | Qualifier ion (m/z) | CE (V) | RF  Lens (V) |
| --- | --- | --- | --- | --- | --- | --- | --- |
| Acetamiprid | 10.19 | 223.0 | 90.1 | 34.8 | 126.0 | 21.6 | 52 |
| Amitraz | 13.70 | 294.1 | 197.2 | 16.0 | 225.1 | 13.7 | 51 |
| Atrazine | 12.66 | 216.1 | 104.0 | 29.4 | 174.1 | 18.2 | 58 |
| Azoxystrobin | 13.22 | 404.2 | 329.1 | 31.1 | 372.2 | 14.2 | 57 |
| Benalaxyl | 14.45 | 326.2 | 148.1 | 21.5 | 294.2 | 10.2 | 49 |
| Bifenazate | 13.78 | 301.2 | 170.1 | 19.6 | 198.1 | 10.2 | 52 |
| Bifenthrin | 14.27 | 440.1 | 181.0 | 41.0 | 166.2 | 10.0 | 57 |
| Bromuconazole | 13.77 | 378.1 | 159.0 | 29.8 | 161.0 | 29.7 | 74 |
| Buprimate | 13.98 | 317.2 | 166.1 | 24.7 | 237.2 | 19.5 | 73 |
| Buprofezin | 15.21 | 306.2 | 116.1 | 16.6 | 201.2 | 12.1 | 45 |
| Carbaryl | 12.13 | 202.1 | 127.0 | 29.6 | 145.0 | 10.2 | 31 |
| Carbendazim | 8.64 | 192.1 | 132.0 | 31.3 | 160.1 | 19.0 | 53 |
| Carbetamide | 11.38 | 237.1 | 118.2 | 13.0 | 192.2 | 10.2 | 37 |
| Carboxin | 12.02 | 236.0 | 87.0 | 24.9 | 143.0 | 15.4 | 50 |
| Chlorantranilprole | 12.92 | 484.3 | 286.0 | 12.7 | 453.1 | 17.4 | 63 |
| Chlorpyriphos | 15.44 | 350.1 | 198.0 | 19.6 | 322.0 | 12.1 | 59 |
| Chloroxuron | 13.84 | 291.1 | 72.0 | 20.8 | 164.1 | 17.1 | 64 |
| Clothianidin | 9.70 | 250.0 | 132.0 | 17.1 | 169.1 | 13.3 | 46 |
| Cyanantraniliprole | 12.16 | 473.0 | 284.0 | 12.5 | 442.0 | 18.0 | 61 |
| Cyazofamid | 14.03 | 325.2 | 108.1 | 13.8 | 261.1 | 10.2 | 51 |
| Cyflufenamid | 14.57 | 413.2 | 241.1 | 23.1 | 295.2 | 15.5 | 63 |
| Cyflumetofen | 14.99 | 465.2 | 173.0 | 23.3 | 249.0 | 13.3 | 59 |
| Cyproconazole | 13.67 | 292.1 | 70.0 | 20.7 | 125.0 | 32.4 | 56 |
| Diazinon | 14.49 | 305.1 | 153.1 | 21.5 | 169.1 | 21.7 | 67 |
| Difenoconazole | 14.78 | 406.2 | 251.0 | 25.2 | 337.2 | 17.3 | 70 |
| Diflubenzuron | 14.14 | 311.1 | 140.9 | 32.4 | 158.1 | 13.9 | 58 |
| Dimethoate | 10.02 | 230.0 | 125.0 | 22.1 | 199.1 | 10.2 | 36 |
| Dimethomorph | 13.63 | 388.2 | 165.1 | 32.2 | 301.2 | 20.7 | 76 |
| Dimoxystrobin | 14.25 | 327.2 | 116.1 | 22.5 | 205.2 | 10.2 | 50 |
| Epoxiconazole | 13.99 | 330.1 | 121.1 | 21.3 | 161.0 | 29.7 | 64 |
| Ethion | 15.30 | 385.1 | 142.8 | 25.5 | 199.0 | 10.2 | 51 |
| Ethofumesate | 13.31 | 287.1 | 121.1 | 16.7 | 259.1 | 10.2 | 54 |
| Etoxazole | 15.59 | 360.2 | 141.0 | 31.0 | 304.2 | 18.0 | 70 |
| Fenamidone | 13.38 | 312.1 | 92.1 | 25.3 | 236.2 | 15.0 | 57 |
| Fenbuconazole | 14.09 | 337.2 | 70.0 | 20.7 | 125.0 | 29.9 | 75 |
| Fenhexamid | 13.90 | 302.2 | 55.0 | 34.1 | 97.1 | 24.1 | 71 |
| Fenpyroximate | 15.76 | 422.3 | 231.1 | 24.6 | 366.2 | 15.2 | 67 |
| Fipronil | 13.33 | 434.7 | 250.2 | 20.0 | 330.2 | 15.0 | 15 |
| Fluopyram | 13.84 | 397.0 | 173.1 | 29.1 | 208.0 | 22.0 | 75 |
| Flutriafol | 12.64 | 302.2 | 70.0 | 19.3 | 123.0 | 28.1 | 61 |
| Hexythiazox | 15.45 | 353.2 | 168.1 | 25.4 | 228.1 | 15.4 | 61 |
| Imidacloprid | 9.67 | 256.1 | 175.1 | 20.1 | 209.2 | 16.8 | 50 |
| Indoxacarb | 14.78 | 528.3 | 249.1 | 16.6 | 293.0 | 13.7 | 71 |
| Isoproturon | 12.78 | 207.1 | 72.0 | 19.4 | 165.1 | 14.9 | 49 |
| Malathion | 13.58 | 331.1 | 99.0 | 22.7 | 127.0 | 12.5 | 41 |
| Mandipropamid | 13.45 | 412.0 | 328.2 | 15.0 | 356.3 | 10.0 | 58 |
| Metaconazole | 14.61 | 320.2 | 70.0 | 23.4 | 125.0 | 38.6 | 60 |
| Metaflumizone | 15.17 | 507.2 | 178.1 | 26.0 | 287.1 | 24.4 | 92 |
| Metalaxyl | 12.77 | 280.2 | 220.2 | 14.2 | 248.2 | 10.2 | 47 |
| Methomyl | 8.59 | 163.0 | 88.1 | 10.2 | 106.1 | 10.2 | 67 |
| Methoxyfenozide | 13.62 | 369.2 | 149.1 | 17.6 | 313.3 | 10.2 | 47 |
| Metrafenone | 14.67 | 409.1 | 209.1 | 12.2 | 226.9 | 19.0 | 58 |
| Metribuzin | 11.71 | 215.1 | 131.0 | 21.8 | 187.2 | 18.7 | 57 |
| Myclobutanil | 13.76 | 289.1 | 70.0 | 19.8 | 125.0 | 32.8 | 61 |
| Novaluron | 14.91 | 493.3 | 141.0 | 41.0 | 158.1 | 19.1 | 75 |
| Oxadiazon | 15.29 | 345.1 | 220.0 | 20.5 | 303.1 | 13.8 | 65 |
| Oxamyl | 8.37 | 237.1 | 72.0 | 10.6 | 90.1 | 10.2 | 54 |
| Penconazole | 14.43 | 284.1 | 69.9 | 18.4 | 159.0 | 30.3 | 61 |
| Phoxim | 14.56 | 299.1 | 77.3 | 30.9 | 129.2 | 10.2 | 53 |
| Pirimicarb | 11.27 | 239.1 | 72.0 | 22.5 | 182.2 | 16.4 | 52 |
| Primiphos | 14.59 | 306.1 | 164.1 | 22.9 | 278.1 | 20.8 | 70 |
| Primiphos-Methyl | 14.59 | 306.2 | 108.1 | 31.3 | 164.1 | 22.6 | 76 |
| Profenofos | 15.12 | 373.0 | 303.0 | 19.0 | 345.0 | 13.3 | 66 |
| Propargite | 15.58 | 368.2 | 175.2 | 16.1 | 231.2 | 10.2 | 46 |
| Propham | 13.20 | 180.0 | 124.1 | 15.5 | 152.1 | 10.2 | 96 |
| Propiconazole | 14.50 | 342.1 | 159.0 | 30.3 | 205.0 | 18.6 | 66 |
| Propoxur | 11.76 | 210.1 | 111.1 | 14.8 | 168.1 | 10.2 | 30 |
| Pyraclostrobin | 14.50 | 388.2 | 163.1 | 24.4 | 194.2 | 12.5 | 52 |
| Pyrazophos | 14.58 | 374.1 | 194.1 | 33.5 | 222.1 | 22.1 | 75 |
| Pyridaben | 15.96 | 365.2 | 147.1 | 25.7 | 309.2 | 13.0 | 46 |
| Pyriproxyfen | 15.30 | 322.1 | 96.1 | 16.9 | 227.2 | 15.1 | 52 |
| Spinetoram | 14.84 | 748.7 | 99.1 | 39.4 | 142.1 | 29.8 | 98 |
| Spinosad A | 14.54 | 732.7 | 99.1 | 38.4 | 142.1 | 28.9 | 96 |
| Spinosad D | 14.82 | 746.7 | 99.1 | 37.9 | 142.1 | 29.0 | 99 |
| Spirodiclofen | 15.74 | 411.2 | 71.1 | 16.1 | 313.2 | 10.2 | 60 |
| Spiromesifen | 15.54 | 371.2 | 255.2 | 31.0 | 273.2 | 11.0 | 76 |
| Spirotetramat | 13.84 | 374.3 | 302.3 | 16.8 | 330.3 | 15.2 | 59 |
| Spiroxamine | 13.52 | 298.3 | 100.2 | 31.0 | 144.2 | 20.4 | 56 |
| Sulfoxaflor | 10.36 | 278.1 | 154.0 | 28.7 | 174.1 | 10.2 | 52 |
| Tebuconazole | 14.40 | 308.2 | 70.0 | 23.1 | 125.0 | 37.0 | 60 |
| Tebufenpyrad | 15.20 | 334.2 | 117.0 | 36.2 | 145.1 | 27.3 | 80 |
| Tebufeozide | 14.17 | 353.2 | 133.0 | 19.2 | 297.3 | 10.2 | 60 |
| Thiacloprid | 10.68 | 253.0 | 99.0 | 42.3 | 126.0 | 21.9 | 59 |
| Thiamethoxam | 8.92 | 292.1 | 181.1 | 23.7 | 211.2 | 12.4 | 40 |
| Thiophanate-methyl | 11.62 | 343.1 | 151.1 | 20.5 | 311.2 | 10.2 | 54 |
| Triadimenol | 13.70 | 296.2 | 70.1 | 10.2 | 227.2 | 10.2 | 44 |
| Trichlorfon | 9.94 | 257.0 | 109.0 | 18.3 | 221.0 | 10.2 | 58 |
| Tricyclazole | 10.94 | 190.0 | 136.0 | 28.8 | 163.1 | 23.2 | 77 |
| Trifloxystrobin | 14.85 | 409.1 | 145.0 | 44.0 | 186.1 | 18.0 | 57 |
| Triticonazole | 13.93 | 318.2 | 70.0 | 19.1 | 249.2 | 17.6 | 61 |
| Zoxsamide | 14.51 | 336.2 | 159.0 | 40.4 | 187.1 | 22.6 | 68 |

Table S2. Linearity range, limit of quantitation (LOQ), intra-day (RSDr), inter-day (RSD_R_) repeatability, matrix effect (%ME), and EU-MRL of the tested pesticides in pepper fruits.

|  | Range  (mg/kg) | LOQ  (mg/kg) | Red Chilli pepper | | |  | Green hot pepper | | |  | Bell pepper | | |  | MRL  (mg/kg) |
| --- | --- | --- | --- | --- | --- | --- | --- | --- | --- | --- | --- | --- | --- | --- | --- |
|  |  |  | RSD_r_ | RSD_R_ | % ME |  | RSD_r_ | RSD_R_ | % ME |  | RSD_r_ | RSD_R_ | % ME |  |  |
| Acetamiprid | 1-100 | 5 | 8.8 | 10.8 | -16.4 |  | 6.1 | 9.7 | -11.8 |  | 7.2 | 13.3 | -9.6 |  | 0.3 |
| Amitraz | 1-100 | 5 | 6.4 | 7.6 | -15.9 |  | 3.8 | 6.8 | -8.8 |  | 5.0 | 9.5 | -8.4 |  | 0.05 |
| Atrazine | 2.5-200 | 10 | 7.9 | 13.3 | -24.0 |  | 4.8 | 9.4 | -12.2 |  | 6.2 | 11.7 | -18.0 |  | 0.05 |
| Azoxystrobin | 1-100 | 5 | 5.6 | 7.7 | -5.1 |  | 3.2 | 6.2 | -7.2 |  | 4.4 | 8.3 | -4.2 |  | 3.0 |
| Benalaxyl | 1-100 | 5 | 7.4 | 10.1 | -11.8 |  | 4.1 | 8.2 | -13.3 |  | 5.7 | 11.0 | -8.5 |  | 0.01 |
| Bifenazate | 2.5-100 | 10 | 8.3 | 11.2 | -18.3 |  | 4.5 | 9.1 | -14.2 |  | 6.4 | 12.4 | -11.0 |  | 3.0 |
| Bifenthrin | 10-200 | 25 | 4.2 | 5.6 | -24.0 |  | 4.2 | 9.0 | -18.8 |  | 6.3 | 12.3 | -14.6 |  | 0.5 |
| Bromuconazole | 5-200 | 10 | 8.3 | 11.0 | -12.1 |  | 5.7 | 13.2 | -11.4 |  | 9.3 | 18.5 | -9.4 |  | 0.01 |
| Buprimate | 5-200 | 10 | 12.1 | 16.7 | -14.9 |  | 7.1 | 13.5 | -9.6 |  | 9.5 | 18.0 | -8.3 |  | 1.5 |
| Buprofezin | 5-200 | 10 | 11.0 | 15.2 | -22.1 |  | 6.4 | 12.3 | -18.7 |  | 8.6 | 16.4 | -13.9 |  | 0.01* |
| Carbaryl | 1-100 | 5 | 4.8 | 6.4 | -16.0 |  | 2.5 | 5.2 | -14.9 |  | 3.7 | 7.1 | -12.6 |  | 0.01 |
| Carbendazim | 2.5-200 | 10 | 5.6 | 7.5 | -17.9 |  | 3.0 | 6.1 | -11.1 |  | 4.3 | 8.3 | -10.5 |  | 0.1* |
| Carbetamide | 1-100 | 5 | 4.3 | 5.7 | -12.3 |  | 2.3 | 4.7 | -7.9 |  | 3.3 | 6.4 | -6.9 |  | 0.01 |
| Carboxin | 1-100 | 5 | 12.4 | 18.1 | -21.2 |  | 10.6 | 14.3 | -23.6 |  | 14.3 | 15.4 | -15.2 |  | 0.03 |
| Chlorantranilprole | 5-200 | 10 | 10.7 | 13.4 | -22.4 |  | 7.9 | 12.0 | -26.4 |  | 8.9 | 16.2 | -16.6 |  | 1.0 |
| Chlorpyriphos | 1-100 | 5 | 7.9 | 12.2 | -5.6 |  | 6.3 | 9.6 | -3.7 |  | 6.7 | 12.0 | -3.2 |  | 0.01* |
| Chloroxuron | 5-200 | 10 | 11.3 | 18.2 | -23.3 |  | 10.0 | 14.2 | -20.2 |  | 9.9 | 17.2 | -14.8 |  | 0.01 |
| Clothianidin | 2.5-200 | 5 | 9.5 | 12.6 | -5.5 |  | 4.8 | 10.3 | -4.3 |  | 7.2 | 14.1 | -3.3 |  | 0.04 |
| Cyanantraniliprole | 1-100 | 5 | 7.2 | 10.5 | -5.1 |  | 4.9 | 8.4 | -4.1 |  | 5.8 | 10.8 | -3.1 |  | 1.5 |
| Cyazofamid | 1-100 | 5 | 6.4 | 10.4 | -12.1 |  | 5.8 | 8.1 | -18.8 |  | 5.6 | 9.7 | -14.9 |  | 0.01 |
| Cyflufenamid | 1-100 | 5 | 9.8 | 14.6 | -14.0 |  | 7.1 | 11.6 | -10.1 |  | 8.1 | 14.8 | -8.2 |  | 0.06 |
| Cyflumetofen | 1-100 | 5 | 11.8 | 17.6 | -6.6 |  | 8.7 | 14.0 | -5.1 |  | 9.8 | 17.8 | -4.0 |  | 0.01 |
| Cyproconazole | 5-200 | 10 | 13.8 | 15.7 | -16.7 |  | 12.5 | 14.7 | -13.3 |  | 12.1 | 16.4 | -10.2 |  | 0.05 |
| Diazinon | 1-100 | 5 | 6.8 | 10.8 | -15.8 |  | 5.8 | 8.4 | -11.4 |  | 5.9 | 10.3 | -9.3 |  | 0.05 |
| Difenoconazole | 5-200 | 10 | 7.9 | 10.5 | -2.6 |  | 4.2 | 8.6 | -4.1 |  | 6.0 | 11.7 | -2.3 |  | 0.9 |
| Diflubenzuron | 1-100 | 5 | 11.5 | 19.6 | -17.1 |  | 11.7 | 15.1 | -11.2 |  | 10.4 | 17.7 | -9.6 |  | 0.01* |
| Dimethoate | 1-100 | 5 | 13.9 | 14.3 | -17.1 |  | 10.4 | 14.2 | -13.4 |  | 11.5 | 18.1 | -10.4 |  | 0.01 |
| Dimethomorph | 5-200 | 10 | 13.0 | 13.7 | -6.0 |  | 10.2 | 13.9 | -3.7 |  | 10.9 | 19.1 | -3.3 |  | 1 |
| Dimoxystrobin | 5-200 | 10 | 4.4 | 5.9 | -17.5 |  | 2.3 | 4.8 | -8.7 |  | 3.4 | 6.5 | -8.9 |  | 0.01 |
| Epoxiconazole | 10-200 | 25 | 7.5 | 13.0 | -15.9 |  | 7.9 | 10.0 | -13.1 |  | 6.9 | 11.6 | -9.9 |  | 0.01 |
| Ethion | 1-100 | 5 | 9.9 | 15.8 | -13.0 |  | 8.6 | 11.3 | -9.8 |  | 8.6 | 10.8 | -7.8 |  | 0.01 |
| Ethofumesate | 10-200 | 25 | 7.2 | 11.8 | -19.8 |  | 6.6 | 9.2 | -17.3 |  | 6.4 | 11.0 | -12.6 |  | 0.03 |
| Etoxazole | 5-200 | 10 | 8.9 | 12.0 | -2.9 |  | 4.9 | 9.8 | -3.8 |  | 6.9 | 13.2 | -2.3 |  | 0.1 |
| Fenamidone | 10-200 | 25 | 4.8 | 6.5 | -14.1 |  | 2.6 | 5.3 | -10.6 |  | 3.7 | 7.2 | -8.4 |  | 0.01 |
| Fenbuconazole | 5-200 | 10 | 8.1 | 11.3 | -11.9 |  | 4.9 | 9.1 | -7.5 |  | 6.4 | 12.1 | -6.6 |  | 0.6 |
| Fenhexamid | 5-200 | 10 | 11.5 | 10.8 | -19.0 |  | 8.4 | 12.0 | -22.9 |  | 9.5 | 17.3 | -18.3 |  | 3 |
| Fenpyroximate | 1-100 | 5 | 11.2 | 18.6 | -21.2 |  | 10.6 | 14.4 | -10.2 |  | 10.0 | 17.2 | -10.7 |  | 0.3 |
| Fipronil | 1-100 | 5 | 8.0 | 10.4 | -13.7 |  | 3.8 | 8.6 | -9.6 |  | 6.0 | 11.9 | -7.9 |  | 0.005 |
| Fluopyram | 1-100 | 5 | 8.0 | 12.5 | -19.1 |  | 6.6 | 10.5 | -22.6 |  | 6.8 | 14.8 | -16.9 |  | 2 |
| Flutriafol | 5-200 | 10 | 7.1 | 9.5 | -1.8 |  | 3.8 | 7.7 | -3.1 |  | 5.4 | 10.5 | -1.7 |  | 1 |
| Hexythiazox | 1-100 | 5 | 5.8 | 7.7 | -15.8 |  | 3.0 | 6.3 | -14.5 |  | 4.4 | 8.6 | -10.3 |  | 0.09 |
| Imidacloprid | 1-100 | 5 | 6.7 | 9.0 | -18.0 |  | 3.6 | 7.3 | -16.1 |  | 5.1 | 9.9 | -11.6 |  | 0.9 |
| Indoxacarb | 5-200 | 10 | 9.7 | 15.9 | -10.5 |  | 9.0 | 12.4 | -7.9 |  | 8.6 | 14.9 | -6.3 |  | 0.3 |
| Isoproturon | 1-100 | 5 | 6.1 | 8.1 | -23.4 |  | 3.2 | 6.6 | -25.4 |  | 4.7 | 9.0 | -16.6 |  | 0.01 |
| Malathion | 1-100 | 5 | 12.1 | 18.3 | -20.0 |  | 9.2 | 13.3 | -16.6 |  | 10.1 | 13.8 | -12.5 |  | 0.02 |
| Mandipropamid | 1-100 | 5 | 5.6 | 7.5 | -15.6 |  | 3.0 | 6.1 | -10.5 |  | 4.3 | 8.3 | -8.9 |  | 1 |
| Metaconazole | 5-200 | 10 | 11.5 | 11.8 | -6.9 |  | 12.2 | 13.3 | -3.8 |  | 10.6 | 17.8 | -3.6 |  | 0.02 |
| Metaflumizone | 2.5-200 | 10 | 5.7 | 9.3 | -11.8 |  | 5.3 | 7.2 | -5.9 |  | 5.0 | 8.7 | -6.0 |  | 1.5 |
| Metalaxyl | 1-100 | 5 | 8.8 | 15.2 | -15.8 |  | 9.2 | 11.7 | -12.8 |  | 8.1 | 13.6 | -9.7 |  | 0.5 |
| Methomyl | 1-100 | 5 | 13.1 | 18.5 | -13.0 |  | 9.5 | 14.0 | -7.6 |  | 10.8 | 14.9 | -7.0 |  | 0.04 |
| Methoxyfenozide | 1-100 | 5 | 8.0 | 10.6 | -21.5 |  | 4.1 | 8.7 | -18.8 |  | 6.1 | 11.9 | -13.7 |  | 2 |
| Metrafenone | 1-100 | 5 | 5.2 | 7.0 | -10.6 |  | 2.8 | 5.7 | -6.7 |  | 4.0 | 7.7 | -5.9 |  | 2 |
| Metribuzin | 2.5-200 | 10 | 9.7 | 15.4 | -14.0 |  | 8.3 | 12.1 | -18.3 |  | 8.4 | 14.8 | -17.4 |  | 0.1 |
| Myclobutanil | 5-200 | 10 | 5.4 | 7.3 | -20.9 |  | 2.9 | 5.9 | -16.6 |  | 4.2 | 8.0 | -12.7 |  | 3 |
| Novaluron | 5-200 | 10 | 4.6 | 6.8 | -8.9 |  | 3.3 | 5.4 | -3.9 |  | 3.8 | 6.9 | -4.4 |  | 0.01 |
| Oxadiazon | 5-200 | 10 | 12.5 | 19.1 | -5.2 |  | 9.7 | 15.1 | -1.8 |  | 10.5 | 18.9 | -2.4 |  | 0.01 |
| Oxamyl | 1-100 | 5 | 14.1 | 15.5 | -9.8 |  | 13.9 | 15.5 | -11.2 |  | 12.7 | 18.4 | -7.1 |  | 0.01 |
| Penconazole | 1-100 | 5 | 11.1 | 14.8 | -16.4 |  | 5.7 | 12.1 | -13.6 |  | 8.5 | 16.5 | -10.2 |  | 0.2 |
| Phoxim | 10-200 | 10 | 6.9 | 10.4 | -14.5 |  | 5.2 | 8.2 | -13.1 |  | 5.8 | 10.5 | -9.4 |  | 0.01 |
| Pirimicarb | 1-100 | 5 | 11.4 | 14.9 | -15.0 |  | 5.6 | 12.2 | -24.3 |  | 8.6 | 16.8 | -16.8 |  | 0.5 |
| Primiphos | 1-100 | 5 | 13.0 | 19.7 | -14.5 |  | 9.9 | 15.6 | -9.7 |  | 10.9 | 19.7 | -8.2 |  | 0.01 |
| Primiphos-Methyl | 1-100 | 5 | 14.4 | 16.2 | -15.9 |  | 10.7 | 14.9 | -11.7 |  | 12.0 | 18.1 | -9.4 |  | 0.01 |
| Profenofos | 1-100 | 5 | 10.8 | 15.5 | -7.3 |  | 7.1 | 12.4 | -3.9 |  | 8.7 | 16.2 | -3.8 |  | 0.01 |
| Propargite | 5-200 | 10 | 6.3 | 10.2 | -9.5 |  | 5.7 | 7.9 | -5.9 |  | 5.5 | 9.6 | -5.3 |  | 0.01* |
| Propham | 5-200 | 10 | 13.1 | 13.3 | -15.2 |  | 9.9 | 13.2 | -11.8 |  | 10.9 | 16.7 | -9.2 |  | 0.01 |
| Propiconazole | 2.5-200 | 10 | 7.4 | 9.9 | -5.3 |  | 3.9 | 8.1 | -4.3 |  | 5.7 | 11.0 | -3.3 |  | 0.01 |
| Propoxur | 1-100 | 5 | 4.7 | 6.4 | -26.3 |  | 2.6 | 5.2 | -19.9 |  | 3.6 | 7.0 | -15.7 |  | 0.005 |
| Pyraclostrobin | 5-200 | 10 | 6.6 | 8.9 | -13.6 |  | 3.6 | 7.2 | -4.9 |  | 5.1 | 9.8 | -6.3 |  | 0.5 |
| Pyrazophos | 10-200 | 10 | 10.5 | 18.3 | 8.1 |  | 11.1 | 14.0 | -6.6 |  | 9.7 | 16.2 | 0.5 |  | 0.01 |
| Pyridaben | 1-100 | 5 | 12.3 | 14.2 | -6.5 |  | 13.1 | 14.3 | -2.7 |  | 11.4 | 17.7 | -3.1 |  | 0.3 |
| Pyriproxyfen | 2.5-200 | 10 | 12.4 | 18.7 | -23.8 |  | 9.3 | 14.8 | -13.9 |  | 10.3 | 18.7 | -12.8 |  | 1 |
| Spinetoram | 1-100 | 5 | 11.0 | 19.1 | -14.2 |  | 11.7 | 14.7 | -22.1 |  | 10.1 | 16.9 | -15.7 |  | 0.4 |
| Spinosad | 1-100 | 5 | 1.5 | 12.1 | -16.3 |  | 1.0 | 4.1 | -13.5 |  | 1.2 | 2.2 | -10.1 |  | 0.6 |
| Spirodiclofen | 10-200 | 25 | 7.8 | 12.9 | -6.7 |  | 7.3 | 10.0 | -3.9 |  | 6.9 | 12.0 | -3.6 |  | 0.2 |
| Spiromesifen | 1-100 | 5 | 3.7 | 5.1 | -8.0 |  | 2.0 | 4.1 | -6.6 |  | 2.9 | 5.6 | -4.9 |  | 0.5 |
| Spirotetramat | 10-500 | 25 | 4.8 | 6.4 | -16.7 |  | 2.6 | 5.2 | -20.1 |  | 3.7 | 7.1 | -9.8 |  | 1 |
| Spiroxamine | 1-100 | 5 | 10.0 | 17.1 | -18.7 |  | 10.2 | 13.2 | -11.6 |  | 9.1 | 15.5 | -10.3 |  | 0.01 |
| Sulfoxaflor | 1-100 | 5 | 7.1 | 11.9 | -24.0 |  | 6.9 | 9.2 | -14.8 |  | 6.4 | 10.9 | -13.2 |  | 0.4 |
| Tebuconazole | 10-200 | 25 | 12.5 | 19.2 | -0.2 |  | 9.9 | 15.1 | -1.6 |  | 10.5 | 18.9 | -0.6 |  | 0.6 |
| Tebufenpyrad | 5-200 | 10 | 14.8 | 15.8 | -7.1 |  | 14.3 | 15.7 | -2.8 |  | 13.3 | 17.9 | -3.4 |  | 0.01 |
| Tebufeozide | 1-100 | 5 | 12.6 | 14.1 | -23.6 |  | 13.2 | 14.8 | -14.7 |  | 11.6 | 19.4 | -13.0 |  | 1.5 |
| Thiacloprid | 1-100 | 5 | 11.9 | 12.1 | -16.9 |  | 12.6 | 13.8 | -10.3 |  | 11.0 | 18.3 | -9.2 |  | 1 |
| Thiamethoxam | 5-200 | 10 | 12.3 | 17.1 | -5.4 |  | 13.2 | 15.4 | -4.1 |  | 11.4 | 19.0 | -3.2 |  | 0.7 |
| Thiophanate-methyl | 5-200 | 10 | 4.0 | 5.4 | -15.7 |  | 2.2 | 4.4 | -10.6 |  | 3.1 | 5.9 | -8.9 |  | 0.1 |
| Triadimenol | 1-100 | 5 | 8.2 | 13.2 | -13.3 |  | 7.2 | 10.3 | -16.2 |  | 7.2 | 12.5 | -14.5 |  | 0.01 |
| Trichlorfon | 1-100 | 5 | 11.8 | 18.2 | 26.7 |  | 9.5 | 14.4 | -21.1 |  | 10.0 | 17.9 | 1.9 |  | 0.01 |
| Tricyclazole | 5-200 | 10 | 4.4 | 5.9 | -18.0 |  | 2.3 | 4.8 | -14.6 |  | 3.4 | 6.5 | -14.5 |  | 0.01 |
| Trifloxystrobin | 1-100 | 5 | 3.6 | 4.8 | -10.9 |  | 1.9 | 3.9 | -6.3 |  | 2.8 | 5.4 | -5.8 |  | 0.4 |
| Triticonazole | 5-200 | 10 | 11.8 | 18.9 | -4.1 |  | 10.3 | 14.8 | -3.1 |  | 10.3 | 18.0 | -2.4 |  | 0.01 |
| Zoxsamide | 5-200 | 10 | 13.3 | 18.2 | -11.6 |  | 13.8 | 15.3 | -7.3 |  | 12.2 | 16.1 | -6.4 |  | 0.02 |

Table S3. Percentage recoveries and relative standard deviation (RSD) of the tested pesticides in pepper fruits.

|  | Red chilli pepper | | | | | | Green hot pepper | | | | | | | Bell pepper | | | | | | | |
| --- | --- | --- | --- | --- | --- | --- | --- | --- | --- | --- | --- | --- | --- | --- | --- | --- | --- | --- | --- | --- | --- |
|  | Spiking level (mg/kg) | | | | | | Spiking level (mg/kg) | | | | | | | Spiking level (mg/kg) | | | | | | | |
|  | 0.01 | | 0.1 | | 1 | | 0.01 | | 0.1 | | 1 | | | 0.01 | | | 0.1 | | | 1 | |
|  | % R | RSD | % R | RSD | % R | RSD | % R | RSD | % R | RSD | % R | RSD | % R | | RSD | % R | | RSD | % R | | RSD |
| Acetamiprid | 93.2 | 2.5 | 96.4 | 2.2 | 97.3 | 1.4 | 94.1 | 7.0 | 95.3 | 4.8 | 93.3 | 6.8 | 88.9 | | 4.9 | 97.8 | | 6.0 | 96.6 | | 5.3 |
| Amitraz | 92.7 | 2.1 | 102.9 | 2.8 | 95.6 | 1.7 | 93.8 | 2.8 | 98.3 | 1.6 | 96.4 | 3.0 | 97.4 | | 1.6 | 99.2 | | 1.4 | 90.9 | | 2.1 |
| Atrazine | 96.3 | 1.6 | 106.4 | 2.6 | 101.7 | 1.9 | 91.7 | 4.9 | 99.1 | 3.4 | 97.1 | 4.8 | 99.2 | | 3.2 | 96.9 | | 3.9 | 91.8 | | 3.7 |
| Azoxystrobin | 98.7 | 1.2 | 97.4 | 2.4 | 101.4* | 1.8* | 93.7 | 2.0 | 95.6 | 1.2 | 93.6 | 2.1 | 96.3 | | 0.9 | 99.3 | | 0.9 | 90.1 | | 1.4 |
| Benalaxyl | 93.9 | 3.6 | 106.9 | 2.8 | 105.5 | 2.6 | 91.1 | 3.1 | 99.0 | 1.4 | 97.0 | 3.6 | 99.9 | | 1.5 | 91.6 | | 0.8 | 91.5 | | 2.3 |
| Bifenazate | 97.3 | 5.8 | 86.7 | 2.9 | 92.8 | 0.4 | 94.3 | 3.1 | 90.5 | 0.8 | 88.7 | 4.3 | 90.6 | | 2.4 | 98.8 | | 1.0 | 85.4 | | 2.5 |
| Bifenthrin | 96.1 | 1.8 | 102.2 | 2.7 | 99.1 | 0.5 | 95.4 | 4.9 | 112.1 | 2.5 | 109.9 | 5.5 | 109.8 | | 3.3 | 93.7 | | 2.8 | 99.4 | | 3.7 |
| Bromuconazole | 98.7 | 4.7 | 93.9 | 4.5 | 102.4 | 1.6 | 88.7 | 4.2 | 96.4 | 0.7 | 94.5 | 6.3 | 97.8 | | 3.1 | 103.9 | | 0.4 | 91.9 | | 3.4 |
| Buprimate | 93.2 | 5.3 | 112.9 | 4.5 | 114.7 | 6.7 | 89.3 | 8.3 | 101.1 | 5.0 | 99.1 | 8.7 | 103.4 | | 4.2 | 104.4 | | 3.9 | 96.4 | | 6.0 |
| Buprofezin | 110.3 | 6.7 | 112.3 | 4.4 | 108.4 | 2.4 | 97.1 | 6.5 | 104.7 | 3.3 | 102.6 | 7.5 | 105.0 | | 4.4 | 97.3 | | 3.5 | 96.4 | | 5.0 |
| Carbaryl | 98.0 | 1.8 | 96.7 | 2.9 | 102.4 | 0.7 | 97.3 | 2.5 | 97.0 | 1.4 | 95.1 | 2.6 | 97.7 | | 1.7 | 98.5 | | 1.7 | 90.7 | | 1.9 |
| Carbendazim | 95.9 | 2.0 | 97.7 | 4.4 | 109.9 | 2.8 | 90.7 | 3.2 | 94.2 | 2.0 | 92.3 | 3.4 | 97.0 | | 1.5 | 107.3 | | 1.4 | 91.7 | | 2.3 |
| Carbetamide | 101.3 | 2.2 | 117.0 | 2.2 | 113.1 | 0.4 | 94.5 | 1.8 | 105.8 | 0.8 | 103.6 | 2.1 | 106.8 | | 1.3 | 108.1 | | 0.9 | 99.8 | | 1.4 |
| Carboxin | 97.4 | 11.3 | 93.7 | 6.0 | 92.8 | 3.8 | 92.2 | 14.1 | 93.0 | 13.3 | 91.1 | 16.7 | 92.5 | | 13.4 | 100.9 | | 12.1 | 87.4 | | 17.2 |
| Chlorantranilprole | 111.2 | 9.6 | 104.5 | 6.5 | 96.8 | 5.4 | 101.7 | 10.4 | 103.1 | 5.5 | 101.0 | 11.7 | 101.4 | | 6.3 | 95.6 | | 5.2 | 93.4 | | 7.8 |
| Chlorpyriphos | 94.9 | 6.3 | 91.3 | 4.2 | 96.2 | 2.0 | 90.6 | 9.7 | 90.9 | 4.3 | 89.1 | 11.7 | 91.6 | | 7.1 | 103.4 | | 5.1 | 87.1 | | 7.7 |
| Chloroxuron | 94.6 | 13.5 | 113.7 | 7.9 | 109.8 | 8.0 | 91.2 | 13.4 | 102.4 | 6.8 | 100.4 | 15.4 | 103.5 | | 7.9 | 100.1 | | 5.8 | 95.9 | | 10.1 |
| Clothianidin | 107.2 | 3.0 | 102.9 | 3.8 | 98.4 | 2.6 | 101.2 | 6.5 | 102.1 | 4.2 | 100.0 | 6.5 | 100.9 | | 4.2 | 92.3 | | 4.7 | 92.3 | | 4.9 |
| Cyanantraniliprole | 100.3 | 3.4 | 98.6 | 6.9 | 113.5 | 6.4 | 94.4 | 6.5 | 96.5 | 4.1 | 94.6 | 6.6 | 99.5 | | 2.9 | 98.0 | | 2.8 | 92.2 | | 4.6 |
| Cyazofamid | 94.4 | 9.9 | 102.7 | 4.9 | 104.4 | 1.9 | 89.8 | 6.3 | 96.2 | 2.2 | 94.3 | 8.1 | 97.5 | | 4.4 | 100.1 | | 2.2 | 91.0 | | 4.9 |
| Cyflufenamid | 94.2 | 9.3 | 110.2 | 6.2 | 103.2 | 3.6 | 90.1 | 9.1 | 100.1 | 4.6 | 98.1 | 10.4 | 100.4 | | 5.9 | 94.9 | | 4.7 | 92.3 | | 7.0 |
| Cyflumetofen | 95.1 | 13.3 | 95.6 | 6.3 | 100.3 | 2.5 | 89.9 | 10.9 | 92.7 | 4.9 | 90.9 | 13.1 | 93.9 | | 7.9 | 101.2 | | 5.7 | 88.3 | | 8.6 |
| Cyproconazole | 95.0 | 15.4 | 109.2 | 11.9 | 101.3 | 11.8 | 92.4 | 17.5 | 100.8 | 9.5 | 98.8 | 19.5 | 100.5 | | 9.8 | 105.3 | | 7.8 | 94.3 | | 13.1 |
| Diazinon | 92.6 | 9.6 | 106.9 | 6.5 | 107.5 | 2.2 | 88.7 | 6.4 | 97.8 | 2.4 | 95.8 | 8.2 | 99.3 | | 4.4 | 104.1 | | 2.4 | 93.3 | | 5.1 |
| Difenoconazole | 93.9 | 3.9 | 94.6 | 3.9 | 95.0 | 3.4 | 89.8 | 4.9 | 92.2 | 2.8 | 90.4 | 5.4 | 92.4 | | 2.7 | 81.7 | | 2.3 | 83.8 | | 3.7 |
| Diflubenzuron | 76.8 | 12.9 | 76.2 | 9.9 | 80.6 | 12.3 | 81.1 | 16.7 | 78.6 | 9.6 | 77.1 | 18.1 | 78.7 | | 8.9 | 104.8 | | 7.6 | 77.3 | | 12.3 |
| Dimethoate | 100.5 | 12.1 | 93.7 | 7.7 | 112.6 | 5.0 | 90.2 | 14.6 | 103.9 | 8.1 | 101.8 | 16.0 | 105.2 | | 9.7 | 98.5 | | 8.9 | 96.8 | | 11.2 |
| Dimethomorph | 98.5 | 11.4 | 103.2 | 8.5 | 99.0 | 8.8 | 93.3 | 14.3 | 98.3 | 8.1 | 96.3 | 15.6 | 98.0 | | 8.2 | 100.3 | | 7.1 | 91.7 | | 10.6 |
| Dimoxystrobin | 91.5 | 2.2 | 102.3 | 2.4 | 110.7 | 0.5 | 87.7 | 1.9 | 99.8 | 0.9 | 97.8 | 2.2 | 101.6 | | 1.3 | 86.6 | | 1.0 | 91.9 | | 1.5 |
| Epoxiconazole | 97.1 | 11.0 | 100.3 | 5.9 | 109.3 | 2.4 | 92.7 | 9.9 | 96.5 | 4.8 | 94.6 | 11.6 | 98.7 | | 7.1 | 91.8 | | 5.5 | 90.5 | | 7.8 |
| Ethion | 88.8 | 9.2 | 95.7 | 6.8 | 97.4 | 6.4 | 82.2 | 11.9 | 89.0 | 6.8 | 87.2 | 12.9 | 90.3 | | 7.2 | 101.9 | | 6.5 | 85.5 | | 9.0 |
| Ethofumesate | 83.4 | 8.9 | 86.7 | 4.7 | 88.1 | 1.9 | 93.9 | 8.2 | 111.3 | 4.0 | 109.0 | 9.6 | 113.5 | | 5.9 | 85.5 | | 4.7 | 101.1 | | 6.4 |
| Etoxazole | 88.6 | 5.9 | 88.4 | 5.0 | 90.3 | 3.6 | 86.4 | 5.7 | 87.4 | 2.9 | 85.7 | 6.5 | 87.6 | | 3.3 | 97.6 | | 2.3 | 82.9 | | 4.3 |
| Fenamidone | 94.3 | 2.2 | 113.3 | 2.1 | 105.2 | 1.2 | 92.2 | 2.5 | 102.8 | 1.3 | 100.7 | 2.8 | 105.0 | | 1.6 | 94.1 | | 1.3 | 95.7 | | 1.9 |
| Fenbuconazole | 88.5 | 6.8 | 98.6 | 4.0 | 98.5 | 0.7 | 84.4 | 5.5 | 91.5 | 2.4 | 89.7 | 6.6 | 92.5 | | 4.1 | 105.2 | | 3.0 | 88.1 | | 4.3 |
| Fenhexamid | 97.4 | 13.1 | 114.6 | 6.3 | 111.0 | 1.9 | 95.5 | 10.3 | 105.1 | 4.5 | 103.0 | 12.5 | 105.8 | | 7.6 | 98.1 | | 5.4 | 96.3 | | 8.1 |
| Fenpyroximate | 92.4 | 9.3 | 89.9 | 7.7 | 92.7 | 10.3 | 88.7 | 15.8 | 113.3 | 9.8 | 103.6 | 16.4 | 89.3 | | 8.8 | 101.6 | | 8.8 | 92.4 | | 11.6 |
| Fipronil | 94.3 | 5.4 | 108.6 | 3.1 | 96.7 | 1.1 | 90.1 | 3.8 | 99.3 | 1.5 | 97.4 | 4.8 | 101.3 | | 2.7 | 101.0 | | 1.6 | 94.0 | | 3.0 |
| Fluopyram | 94.9 | 8.8 | 94.2 | 4.8 | 105.1 | 3.8 | 92.2 | 8.3 | 99.4 | 4.1 | 97.4 | 9.6 | 100.1 | | 5.2 | 106.1 | | 3.9 | 94.2 | | 6.3 |
| Flutriafol | 102.1 | 3.5 | 115.2 | 4.7 | 113.7 | 2.9 | 97.8 | 4.3 | 106.5 | 2.4 | 104.4 | 4.7 | 107.5 | | 2.4 | 91.7 | | 2.0 | 97.5 | | 3.2 |
| Hexythiazox | 90.6 | 2.1 | 93.3 | 3.1 | 96.6 | 1.0 | 91.1 | 3.3 | 92.2 | 2.0 | 90.4 | 3.5 | 92.7 | | 2.3 | 100.3 | | 2.3 | 87.2 | | 2.5 |
| Imidacloprid | 93.5 | 3.2 | 103.9 | 2.5 | 111.9 | 1.9 | 88.7 | 4.0 | 96.3 | 2.3 | 94.4 | 4.4 | 99.0 | | 2.5 | 106.8 | | 2.3 | 93.3 | | 3.0 |
| Indoxacarb | 106.6 | 14.0 | 112.2 | 8.3 | 110.3 | 3.1 | 101.3 | 11.2 | 106.8 | 5.0 | 104.6 | 13.6 | 107.0 | | 7.9 | 102.5 | | 5.5 | 98.8 | | 8.8 |
| Isoproturon | 97.2 | 2.3 | 108.5 | 3.6 | 101.3 | 1.1 | 96.7 | 3.6 | 102.6 | 2.1 | 100.5 | 3.7 | 101.9 | | 2.4 | 97.4 | | 2.5 | 94.0 | | 2.7 |
| Malathion | 86.9 | 12.6 | 105.3 | 6.6 | 109.1 | 4.4 | 82.5 | 12.1 | 93.9 | 6.0 | 92.0 | 14.0 | 96.6 | | 8.1 | 101.1 | | 6.4 | 90.6 | | 9.3 |
| Mandipropamid | 92.3 | 1.8 | 110.6 | 2.7 | 114.9 | 1.3 | 86.8 | 3.3 | 98.7 | 2.1 | 96.7 | 3.4 | 101.6 | | 2.1 | 93.8 | | 2.3 | 93.2 | | 2.5 |
| Metaconazole | 90.9 | 10.2 | 91.5 | 7.8 | 98.4 | 11.1 | 93.7 | 13.1 | 92.6 | 11.8 | 90.7 | 6.9 | 93.4 | | 10.7 | 107.2 | | 11.2 | 89.1 | | 13.8 |
| Metaflumizone | 102.0 | 7.7 | 105.2 | 3.5 | 112.8 | 1.1 | 98.3 | 6.0 | 107.0 | 2.6 | 104.8 | 7.3 | 107.7 | | 4.4 | 100.9 | | 3.1 | 99.0 | | 4.8 |
| Metalaxyl | 100.2 | 10.5 | 108.9 | 5.4 | 103.1 | 2.7 | 91.2 | 12.7 | 100.1 | 7.1 | 98.0 | 13.9 | 100.3 | | 9.1 | 104.0 | | 8.6 | 93.7 | | 9.9 |
| Methomyl | 104.4 | 10.8 | 114.8 | 5.4 | 106.7 | 2.8 | 101.1 | 13.2 | 108.0 | 7.4 | 105.8 | 14.5 | 107.3 | | 9.4 | 102.2 | | 9.0 | 99.1 | | 10.2 |
| Methoxyfenozide | 87.3 | 2.2 | 101.1 | 2.3 | 103.0 | 2.4 | 83.3 | 5.4 | 92.2 | 3.6 | 90.4 | 5.3 | 94.0 | | 3.3 | 102.2 | | 3.9 | 88.6 | | 4.0 |
| Metrafenone | 95.8 | 2.7 | 111.8 | 3.7 | 106.7 | 1.2 | 89.8 | 2.7 | 100.8 | 1.4 | 98.8 | 3.0 | 101.6 | | 1.7 | 104.7 | | 1.3 | 95.3 | | 2.0 |
| Metribuzin | 95.3 | 10.5 | 93.7 | 8.5 | 89.6 | 5.2 | 92.7 | 11.0 | 104.7 | 5.7 | 102.6 | 12.4 | 106.7 | | 6.9 | 85.6 | | 5.5 | 95.6 | | 8.3 |
| Myclobutanil | 81.1 | 1.8 | 83.9 | 3.1 | 82.0 | 2.8 | 85.5 | 3.1 | 84.7 | 1.9 | 83.0 | 3.2 | 83.8 | | 1.4 | 99.1 | | 1.3 | 80.3 | | 2.2 |
| Novaluron | 88.0 | 2.3 | 106.8 | 2.5 | 111.3 | 2.4 | 83.3 | 3.7 | 95.0 | 2.3 | 93.1 | 3.9 | 97.9 | | 2.1 | 91.6 | | 2.1 | 89.9 | | 2.7 |
| Oxadiazon | 88.5 | 12.3 | 93.9 | 7.6 | 97.4 | 7.3 | 84.7 | 13.1 | 89.3 | 6.9 | 87.5 | 14.8 | 90.6 | | 7.9 | 97.7 | | 6.2 | 85.3 | | 9.9 |
| Oxamyl | 100.3 | 12.8 | 98.9 | 10.9 | 105.5 | 13.6 | 92.7 | 11.1 | 95.8 | 12.9 | 93.9 | 8.7 | 97.4 | | 11.8 | 97.4 | | 11.7 | 90.5 | | 15.5 |
| Penconazole | 93.2 | 6.0 | 102.3 | 5.2 | 103.8 | 3.2 | 90.1 | 7.4 | 96.2 | 4.1 | 94.3 | 8.1 | 97.3 | | 4.7 | 96.0 | | 4.3 | 90.4 | | 5.6 |
| Phoxim | 90.3 | 5.5 | 90.9 | 3.2 | 93.0 | 2.8 | 85.6 | 6.4 | 88.2 | 3.5 | 86.5 | 7.1 | 88.8 | | 4.1 | 98.8 | | 3.6 | 84.1 | | 4.9 |
| Pirimicarb | 100.5 | 5.1 | 102.2 | 4.3 | 105.6 | 1.9 | 92.8 | 7.4 | 97.5 | 4.4 | 95.5 | 7.8 | 98.7 | | 5.2 | 96.9 | | 5.2 | 91.5 | | 5.7 |
| Primiphos | 89.8 | 10.7 | 104.8 | 7.7 | 94.8 | 4.9 | 83.4 | 14.0 | 94.1 | 8.0 | 92.2 | 15.1 | 96.9 | | 9.3 | 93.4 | | 8.9 | 89.4 | | 10.7 |
| Primiphos-Methyl | 93.9 | 11.9 | 95.0 | 7.1 | 95.0 | 6.4 | 89.2 | 13.3 | 92.1 | 8.7 | 90.3 | 11.6 | 92.3 | | 9.8 | 100.5 | | 9.1 | 87.4 | | 11.6 |
| Profenofos | 88.3 | 10.3 | 90.7 | 5.3 | 92.4 | 3.3 | 82.6 | 8.7 | 99.2 | 4.0 | 97.2 | 10.4 | 102.0 | | 5.9 | 84.5 | | 4.1 | 91.8 | | 6.8 |
| Propargite | 88.0 | 9.4 | 103.2 | 6.3 | 94.6 | 1.5 | 85.6 | 6.2 | 94.4 | 2.3 | 92.5 | 7.9 | 100.0 | | 4.5 | 96.0 | | 2.5 | 92.3 | | 4.9 |
| Propham | 99.9 | 11.3 | 98.4 | 8.4 | 100.9 | 4.9 | 90.3 | 13.9 | 94.3 | 7.8 | 92.5 | 15.2 | 95.3 | | 9.2 | 99.4 | | 8.5 | 89.2 | | 10.6 |
| Propiconazole | 96.9 | 3.2 | 104.3 | 3.9 | 108.2 | 2.0 | 91.1 | 4.7 | 97.7 | 2.8 | 95.7 | 5.0 | 99.4 | | 3.0 | 96.0 | | 3.0 | 92.0 | | 3.5 |
| Propoxur | 99.2 | 1.7 | 93.1 | 3.0 | 105.6 | 1.7 | 96.4 | 2.7 | 94.8 | 1.6 | 92.9 | 2.8 | 96.6 | | 1.5 | 89.0 | | 1.4 | 88.4 | | 2.0 |
| Pyraclostrobin | 93.6 | 3.0 | 82.8 | 3.5 | 93.9 | 2.1 | 90.6 | 4.1 | 86.7 | 2.3 | 85.0 | 4.4 | 87.8 | | 2.5 | 96.7 | | 2.3 | 82.0 | | 3.1 |
| Pyrazophos | 97.1 | 16.8 | 81.8 | 8.9 | 85.3 | 3.0 | 102.2 | 14.5 | 92.0 | 6.8 | 90.2 | 17.2 | 90.3 | | 10.6 | 93.5 | | 8.1 | 93.9 | | 11.4 |
| Pyridaben | 89.6 | 8.7 | 87.8 | 10.2 | 85.7 | 15.0 | 94.4 | 12.8 | 103.7 | 13.8 | 101.8 | 10.6 | 89.9 | | 11.0 | 85.0 | | 12.3 | 91.7 | | 15.2 |
| Pyriproxyfen | 83.5 | 11.8 | 81.4 | 6.9 | 86.4 | 3.5 | 78.8 | 12.5 | 80.1 | 6.6 | 78.5 | 14.2 | 81.0 | | 8.7 | 115.8 | | 7.6 | 80.7 | | 9.7 |
| Spinetoram | 88.2 | 10.7 | 91.1 | 8.1 | 93.8 | 5.4 | 82.4 | 12.1 | 86.8 | 8.5 | 85.0 | 8.8 | 87.8 | | 10.9 | 83.0 | | 9.3 | 80.8 | | 12.4 |
| Spinosad A | 83.3 | 10.1 | 85.5 | 6.2 | 91.1 | 3.7 | 81.1 | 11.9 | 82.8 | 6.9 | 81.1 | 12.8 | 82.3 | | 6.9 | 87.9 | | 6.3 | 77.4 | | 8.8 |
| Spirodiclofen | 90.7 | 8.8 | 88.4 | 6.5 | 90.0 | 3.6 | 86.6 | 9.6 | 87.5 | 5.1 | 85.8 | 10.8 | 87.7 | | 6.3 | 87.8 | | 5.4 | 81.3 | | 7.4 |
| Spiromesifen | 87.9 | 1.8 | 86.8 | 3.8 | 95.6 | 0.6 | 82.1 | 1.5 | 84.5 | 0.7 | 82.8 | 1.8 | 86.3 | | 1.0 | 97.3 | | 0.7 | 82.2 | | 1.1 |
| Spirotetramat | 90.0 | 1.8 | 101.5 | 4.0 | 104.4 | 0.7 | 93.8 | 2.5 | 97.7 | 1.5 | 95.7 | 2.6 | 98.6 | | 1.7 | 83.9 | | 1.7 | 89.1 | | 1.9 |
| Spiroxamine | 82.7 | 12.7 | 84.2 | 9.2 | 80.9 | 9.8 | 80.5 | 7.9 | 82.4 | 7.5 | 80.7 | 4.4 | 81.7 | | 7.7 | 100.2 | | 5.8 | 78.9 | | 10.4 |
| Sulfoxaflor | 100.4 | 7.8 | 103.1 | 5.7 | 105.4 | 3.6 | 96.6 | 9.1 | 99.9 | 5.0 | 97.9 | 10.1 | 100.6 | | 6.0 | 94.9 | | 5.3 | 92.8 | | 7.0 |
| Tebuconazole | 94.2 | 6.8 | 94.8 | 8.0 | 95.3 | 9.8 | 97.7 | 15.2 | 96.2 | 10.0 | 94.3 | 11.2 | 95.7 | | 8.4 | 87.8 | | 9.3 | 87.5 | | 11.1 |
| Tebufenpyrad | 88.9 | 16.9 | 89.0 | 9.6 | 90.5 | 9.7 | 81.7 | 9.8 | 85.4 | 11.6 | 83.7 | 12.7 | 86.1 | | 13.0 | 100.6 | | 11.6 | 82.2 | | 15.7 |
| Tebufeozide | 91.2 | 15.1 | 108.3 | 10.0 | 108.7 | 8.0 | 88.2 | 11.1 | 98.2 | 10.7 | 96.3 | 7.1 | 99.9 | | 12.2 | 103.0 | | 11.2 | 93.4 | | 14.4 |
| Thiacloprid | 98.2 | 10.1 | 101.8 | 6.3 | 104.6 | 8.5 | 91.1 | 11.1 | 96.5 | 11.3 | 94.5 | 9.7 | 97.7 | | 11.7 | 94.3 | | 11.8 | 90.3 | | 14.1 |
| Thiamethoxam | 96.2 | 14.6 | 95.8 | 8.4 | 96.7 | 10.5 | 92.4 | 9.3 | 94.1 | 8.7 | 92.2 | 6.1 | 94.2 | | 11.5 | 93.0 | | 10.5 | 87.4 | | 14.4 |
| Thiophanate-methyl | 98.4 | 1.7 | 93.7 | 3.5 | 94.9 | 0.7 | 91.3 | 1.8 | 92.5 | 0.9 | 90.7 | 2.0 | 92.6 | | 1.2 | 94.6 | | 1.0 | 86.3 | | 1.4 |
| Triadimenol | 85.3 | 10.0 | 95.7 | 8.0 | 102.6 | 2.6 | 88.7 | 9.1 | 92.2 | 4.4 | 90.3 | 10.6 | 93.9 | | 6.3 | 97.4 | | 4.9 | 87.8 | | 7.0 |
| Trichlorfon | 103.1 | 10.9 | 94.4 | 8.3 | 99.7 | 6.4 | 98.6 | 13.1 | 96.5 | 7.3 | 94.6 | 4.4 | 96.8 | | 8.1 | 100.3 | | 7.1 | 90.6 | | 9.9 |
| Tricyclazole | 98.1 | 2.0 | 109.4 | 1.2 | 101.3 | 0.6 | 92.7 | 2.0 | 101.0 | 1.0 | 99.0 | 2.3 | 100.7 | | 1.4 | 95.6 | | 1.2 | 92.8 | | 1.6 |
| Trifloxystrobin | 94.3 | 1.9 | 98.9 | 1.1 | 100.3 | 0.4 | 89.8 | 1.1 | 94.3 | 0.3 | 92.4 | 1.5 | 95.2 | | 0.8 | 97.2 | | 0.3 | 88.7 | | 0.9 |
| Triticonazole | 102.3 | 14.4 | 98.7 | 8.5 | 99.8 | 3.2 | 93.8 | 13.7 | 96.2 | 6.8 | 94.3 | 5.8 | 96.6 | | 9.8 | 100.6 | | 7.9 | 90.3 | | 10.7 |
| Zoxsamide | 93.6 | 14.1 | 102.4 | 12.3 | 103.5 | 11.5 | 96.6 | 6.1 | 99.5 | 4.7 | 97.5 | 5.9 | 99.9 | | 12.2 | 96.1 | | 11.9 | 92.9 | | 15.4 |

*Recovery and precision at spiking level of 3 mg/kg
